# Magic-angle-spinning NMR structure of the kinesin-1 motor domain assembled with microtubules reveals the elusive neck linker orientation

**DOI:** 10.1038/s41467-022-34026-w

**Published:** 2022-11-10

**Authors:** Chunting Zhang, Changmiao Guo, Ryan W. Russell, Caitlin M. Quinn, Mingyue Li, John C. Williams, Angela M. Gronenborn, Tatyana Polenova

**Affiliations:** 1 Department of Chemistry and Biochemistry, University of Delaware, Newark, DE USA; 2 Department of Molecular Medicine, Beckman Research Institute of City of Hope, Duarte, CA USA; 3 Department of Structural Biology, University of Pittsburgh School of Medicine, Pittsburgh, PA USA; 4 Pittsburgh Center for HIV Protein Interactions, University of Pittsburgh School of Medicine, 1051 Biomedical Science Tower 3, 3501 Fifth Ave., Pittsburgh, PA 15261 USA

**Keywords:** Solid-state NMR, Supramolecular assembly, Cytoskeletal proteins

## Abstract

Microtubules (MTs) and their associated proteins play essential roles in maintaining cell structure, organelle transport, cell motility, and cell division. Two motors, kinesin and cytoplasmic dynein link the MT network to transported cargos using ATP for force generation. Here, we report an all-atom NMR structure of nucleotide-free kinesin-1 motor domain (apo-KIF5B) in complex with paclitaxel-stabilized microtubules using magic-angle-spinning (MAS) NMR spectroscopy. The structure reveals the position and orientation of the functionally important neck linker and how ADP induces structural and dynamic changes that ensue in the neck linker. These results demonstrate that the neck linker is in the undocked conformation and oriented in the direction opposite to the KIF5B movement. Chemical shift perturbations and intensity changes indicate that a significant portion of ADP-KIF5B is in the neck linker docked state. This study also highlights the unique capability of MAS NMR to provide atomic-level information on dynamic regions of biological assemblies.

## Abstract (web summary)

In this work the authors report the structure of nucleotide-free kinesin-1 motor domain (apo-KIF5B) in complex with paclitaxel-stabilized microtubules using magic-angle-spinning (MAS) NMR spectroscopy. The study provides insights into the dynamic changes under which the neck linker goes upon binding to ADP.

## Introduction

Microtubules (MTs) are cytoskeleton filaments composed of α/β tubulin dimers. MTs are essential to all dividing and differentiated eukaryotic cells and provide the scaffold required for a variety of cellular activities including cell structure maintenance, intracellular transport of organelles and cargos, cell migration and cell division^1–4^. The kinesin superfamily of proteins (KIFs) are motors that transport cargos and organelles along microtubules to specific destinations using ATP hydrolysis as the energy source. Kinesin motors also play important roles in cell division, especially in the formation of mitotic spindles^5,6^. Not surprisingly, mutations in kinesins lead to a host of diseases and given their critical role in cell division, kinesins are also potential targets for cancer therapeutics^7,8^.

Kinesin-1 (also referred to as conventional kinesin) is the founding member of the kinesin superfamily. Among the kinesin-1 family members, KIF5B is ubiquitously expressed, while KIF5A and KIF5C are exclusively expressed in neurons^9^. The mechanism of kinesin’s processive movement along MTs has been extensively studied by optical trapping and other biophysical methods, and the asymmetric hand-over-hand model is generally accepted^10,11^. In this model, the action in one motor would be transmitted to the other motor for processive movements of dimeric kinesin-1 motors^12^ along MTs, where the neck linker region in each kinesin monomer plays a critical role in this process^13^. Kinesin adopts three chemical states during the catalytic cycle: the apo-, ADP-, and ATP-states^14–16^. At the beginning of the cycle, apo-kinesin binds to MTs while waiting for ATP. Upon ATP binding to the leading head in ATP-kinesin, the trailing head is rotated towards the processive direction driven by the ATP hydrolysis and becomes the new leading head. During this stepping process, the neck linker of the ATP-bound head is docked and the trailing head detaches from MT with the Pi release (ADP-kinesin)^10,11,17,18^. Consequently, the molecular structures of kinesin in each catalytic state are different. Specifically, kinesin in apo- and ATP-states exhibits high-affinity binding to MTs, with the neck linker undocked and docked, respectively. In contrast, the ADP-state of kinesin has a much lower binding affinity to MTs, with the neck linker existing in a two-state equilibrium between the undocked and the docked position^19–21^. The neck linker region, comprising the C-terminal residues K323-L335, has been reported to undergo major conformational changes between the different states and is suggested to be the conduit for informing one motor subunit about the state of the other, generating force and directionality^19,22–25^.

Recent studies determined the structures of multiple kinesin motors bound to microtubules^26–31^. Several high-resolution structural models of the KIF5B motor domain in different states, including free KIF5B, KIF5B in complex with a tubulin dimer^32^, and KIF5B bound with polymerized MTs, have been determined by X-ray crystallography and cryo-EM, at resolutions of 1.8 Å (PDB ID: 1BG2), 2.2 Å (PDB ID: 4LNU), 6 Å (PDB ID: 3J8X) and 3.6 Å (EMD: 21919), respectively^20,32–35^. However, a structure of KIF5B with a well-defined neck linker in complex with polymerized MTs had not been determined to date. Magic-angle-spinning (MAS) NMR is a powerful technique for atomic-resolution structure determinations of proteins in biological assemblies and yields residue-specific structural and dynamics information. Previous studies have demonstrated that accurate and precise protein structures can be determined by integrating NMR experimental distance restraints with low-to-medium resolution cryo-EM density maps for large biomolecular complexes^36–40^. Using this integrated approach, fewer restraints are required and the structures can be refined to higher precision and accuracy when cryo-EM maps are available.

Here, we report the all-atom NMR structure of apo-KIF5B in complex with polymerized microtubules, determined by MAS NMR. The structure was calculated based on 1,339 non-redundant MAS NMR-derived ^13^C-^13^C distance and 494 torsion angle restraints and the medium-resolution (6 Å) electron density map of apo-KIF5B bound to microtubules (EMD-6187)^34^. The functionally important neck linker region and the nucleotide-binding site are clearly defined in the structure. Remarkably, the structure reveals the position and orientation of the flexible neck linker in the undocked position that is missing in previous studies. Pronounced structural and dynamic changes were observed in the apo- and ADP- states of KIF5B bound to microtubules. More generally, beyond the intriguing details of the current structure of a microtubule-associated protein bound to polymerized MTs, our results emphasize the power of integrating MAS NMR with medium-resolution cryo-EM maps.

## Results

### Resonance assignments and distance restraints

**Fig. 1:**
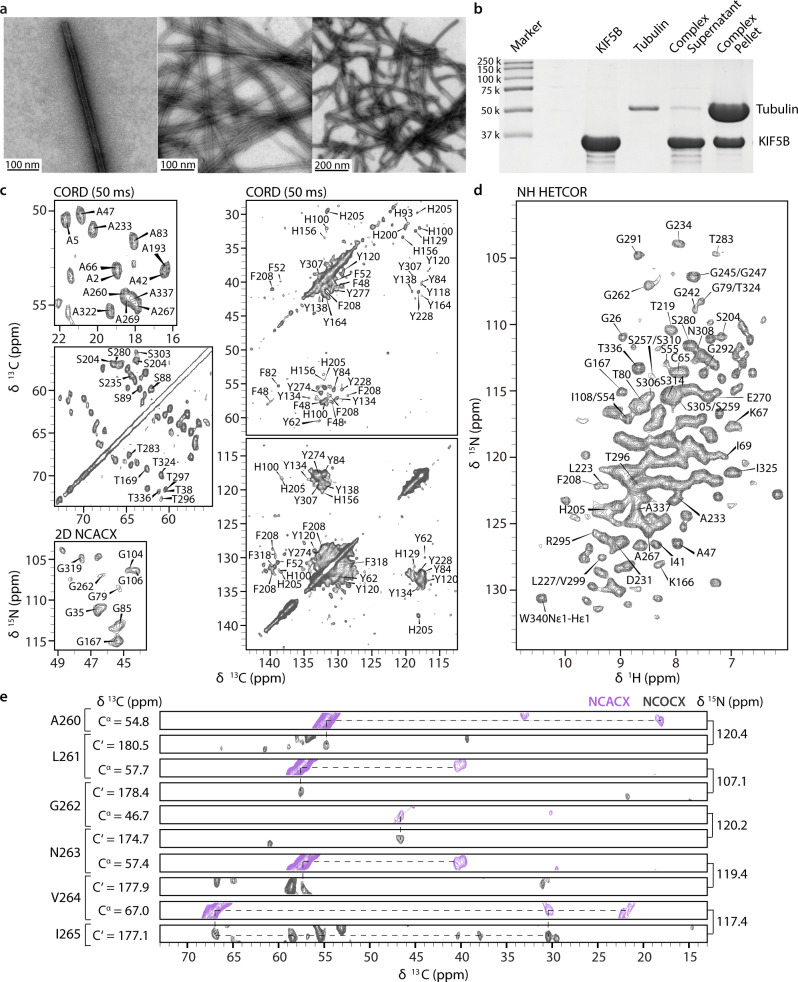
Characterization and MAS NMR spectra of KIF5B bound to MTs. **a** TEM images of the NMR sample before (left, middle) and after (right) MAS NMR experiments. Similar results were reproduced in three independent experiments. **b** SDS-PAGE  of the KIF5B/MT complex. The co-sedimentation assay conditions are detailed in Methods. This experiment was replicated three times with similar results. **c** Characteristic Ala (top left), Ser/Thr (middle left), Gly (bottom left) and aromatic (right) regions of the CORD and NCACX spectra of U-^13^C,^15^N-KIF5B bound to MTs. **d** 2D ^1^H-^15^N HETCOR spectra of U-^2^H,^13^C,^15^N-KIF5B bound to MTs. **e** Backbone walk for the A260-I265 stretch extracted from 3D NCACX (purple) and NCOCX (gray) spectra of U-^13^C,^15^N-KIF5B bound to MTs. Selected assignments are labeled in each spectrum. Source data are provided as a Source Data file.

Negatively stained TEM images of the NMR sample containing U-^13^C,^15^N-KIF5B bound to polymerized MTs are shown in Fig. 1a. To corroborate that MAS NMR conditions do not interfere with the sample morphology, TEM images were collected before and after the experiments and there are no indications of depolymerization or microtubule filament disruption after spinning the sample at a MAS frequency of 14 kHz for one month. Complex formation of U-^13^C,^15^N-KIF5B/MT was confirmed by co-sedimentation and SDS-PAGE analysis (Fig. 1b), confirming specific binding between KIF5B and MTs. The TEM and co-sedimentation assay results are highly reproducible over multiple samples.

KIF5B/MT complexes yield remarkably well-resolved 2D ^13^C-detected CORD, NCACX and ^1^H-detected NH HETCOR spectra. The expansions in characteristic regions of the spectra are provided in Fig. 1c,d and Supplementary Fig. 2. The overall number of cross-peaks in the spectra is consistent with most expected short-range intra-residue correlations being present. The linewidths of individual cross peaks are 0.2–0.5 ppm, indicating that KIF5B adopts a well-ordered structure when bound to MTs.

A major challenge for resonance assignments of KIF5B bound to MTs is the large size of the complex (349 aa, 39.3 kDa) and the low concentration of the labeled component (~9%) in the NMR sample, resulting in spectral congestion and low sensitivity. To alleviate sensitivity limitations, we recorded spectra using a CPMAS CryoProbe^41^, which yields 3-4 fold sensitivity enhancements compared to conventional MAS NMR probes and employed nonuniform sampling (NUS)^42–44^. Overall, we recorded seven 2D CORD spectra with different mixing times on four sets of samples as well as two 2D NCACX, 3D NCACX and NCOCX spectra on two sets of samples (Supplementary Table 1). From these spectra, 2783 cross peaks were assigned (Table 1). ^13^C and ^15^N chemical shifts were assigned for 289 residues, using 2D and 3D homonuclear and heteronuclear correlation spectra. Selected strips, extracted from 3D NCACX and NCOCX spectra, showing a backbone walk for residues A260 to V264, are shown in Fig. 1e. All assignments are summarized in Supplementary Table 2 and Supplementary Fig. 10.

**Table 1:** Summary of samples and the number of assigned peaks

ID	Sample	Assigned peak type	No. assigned peaks^a^
I	U-^13^C,^15^N	Intra-residue	1718
		Sequential	285
		Medium range (1 < |i-j | <5)	4
		Long range (|i-j | ≥5)	3
		Ambiguous	230
II	[1,6-^13^C-glucose], U-^15^N	Intra-residue	152
		Sequential	21
		Medium range (1 < |i-j | <5)	32
		Long range (|i-j | ≥5)	63
		Ambiguous	27
III	[2-^13^C-glucose], U-^15^N	Intra-residue	128
		Sequential	16
		Medium range (1 < |i-j | <5)	17
		Long range (|i-j | ≥5)	76
		Ambiguous	11
Total Assigned Peaks			2783

^a^Cross peaks present in different experiments are counted only once.

**Fig. 2:**
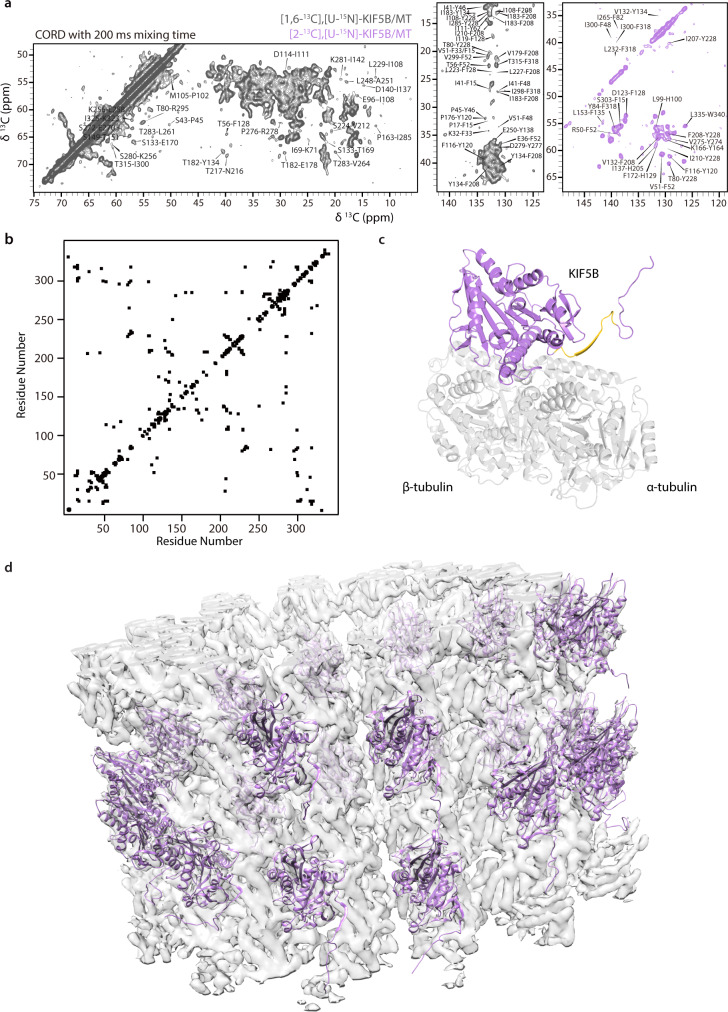
MAS NMR spectra and structure of KIF5B bound to MTs. **a** Expansions of 2D CORD spectra (200 ms mixing time) of [1,6-^13^C-glucose,U-^15^N]-KIF5B/MT (gray) and [2-^13^C-glucose,U-^15^N]-KIF5B/MT (purple). Inter-residue correlations are labeled. **b** A matrix representation of assigned inter-residue contacts generated from the MAS NMR distance restraints. **c** Expansion of a single KIF5B (purple, with the neck linker colored in yellow) bound to a tubulin dimer (gray). **d** Side view of 22 KIF5B structures (purple) docked onto microtubules (gray).

^13^C-^13^C correlations were extracted from 2D CORD spectra of [1,6-^13^C-glucose, U-^15^N]-KIF5B/MT and [2-^13^C-glucose,U-^15^N]-KIF5B/MT samples. As shown in Fig. 2a and Supplementary Fig. 3, a large number of well-resolved cross peaks, corresponding to backbone-to-backbone, backbone-to-sidechain and sidechain-to-sidechain contacts are present. Figure 2b displays the 2D contact map. These correlations were converted to distance restraints with bounds of 1.5–6.5 Å (4.0 ± 2.5 Å) and 2.0–7.2 Å (4.6 ± 2.6 Å) for intra- and inter-residue restraints, respectively, consistent with our previous studies^39,45^. In total, 1339 non-redundant distances were extracted, 1140 of which are unambiguous. Of these, 209 are sequential, inter-residue medium-range (1 < |*i*-*j* | <5) and long-range (|*i*-*j* | ≥5) distances. A summary of all restraints is provided in Table 2. The number of restraints per residue mapped on the 3D structure is shown in Supplementary Fig. 4.

**Table 2:** NMR distance and dihedral constraints

Distance constraints	
Unambiguous	1146
Intra-residue	937
Inter-residue	209
Sequential (|*i*-*j* | = 1)	55
Medium-range (1 ≤ |*i*-*j* | <5)	43
Long-range (|*i*-*j* | ≥ 5) (sidechain-sidechain)	111 (56)
Ambiguous	193
Total ^13^C-^13^C restraints	1339
Restraints/Residue	3.85
Summary of dihedral angle restraints	
ϕ	247
ψ	247
Structure statistics from 22 lowest energy subunits	
Violations (mean ± s.d.)	
Distance restraints ≥ 7.2 Å (Å)	0.168 ± 0.001
Dihedral angle restraints ≥ 5° (°)	1.421 ± 0.077
Max. distance restraint violation^a^ (Å)	0.855
Max. dihedral angle restraint violation (°)	13.230
Deviations from idealized geometry	
Bond lengths (Å)	0.005 ± 0.010
Bond angles (°)	0.667 ± 0.388
Impropers (°)	0.756 ± 1.152
Average pairwise r.m.s.d.^a^ (Å)	
Heavy	1.24 ± 0.09
Backbone (N, Cα, C)	0.89 ± 0.09

^a^Pairwise r.m.s. deviation was calculated among 22-member ensemble.

### Structure of KIF5B bound to polymerized microtubules

The secondary structure of KIF5B bound to polymerized MTs predicted from NMR chemical shifts is consistent with the cryo-EM structure of KIF5B bound to MTs (PDB ID: 3J8X^34^) and the delineation of secondary structure elements is provided in Supplementary Fig. 5.

The all-atom NMR structure of apo-KIF5B bound to MTs was determined by the integration of experimental MAS NMR-derived restraints and a medium-resolution cryo-EM density map (EMD-6187; 6 Å resolution). The structures of a single KIF5B chain bound to a tubulin dimer unit in the overall complex and of 22 KIF5B chains docked to microtubules are shown in Fig. 2c and d, respectively.

The protocol for structure determination entailed as the starting structure a single-chain generated by simulated annealing in Xplor-NIH^46–48^. Next, lowest energy single-chain structures were batch docked into the density (EMD-6187, 6 Å) using 22 unique positions, identified on the basis of cross-correlation values of each fit. Joint refinement of the 22-subunit assembly was carried out using the MAS NMR-derived restraints and the cryo-EM density. For the functionally critical neck-linker region a final round of refinement was performed followed by an all-atom minimization. The precision of the 10 lowest-energy conformer ensemble for the KIF5B chains, measured by pairwise atomic backbone rmsd, is 1.2 ± 0.1 Å. All structure statistics are presented in Table 2. The structure of apo-KIF5B was validated using structures of kinesin-1 in different nucleotide states as the initial models, namely ADP-state (PDB 4HNA^32^) and ATP-state kinesin (PDB 3J8Y) coordinates. The KIF5B structures were then calculated and refined with MAS NMR restraints. The resulting structures are identical, the same as our structure calculated from the initial model of nucleotide-free KIF5B and different from the starting coordinates (Supplementary Fig. 6). These structure validations ascertain that the structures of KIF5B are defined by experimental MAS NMR restraints.

**Fig. 3:**
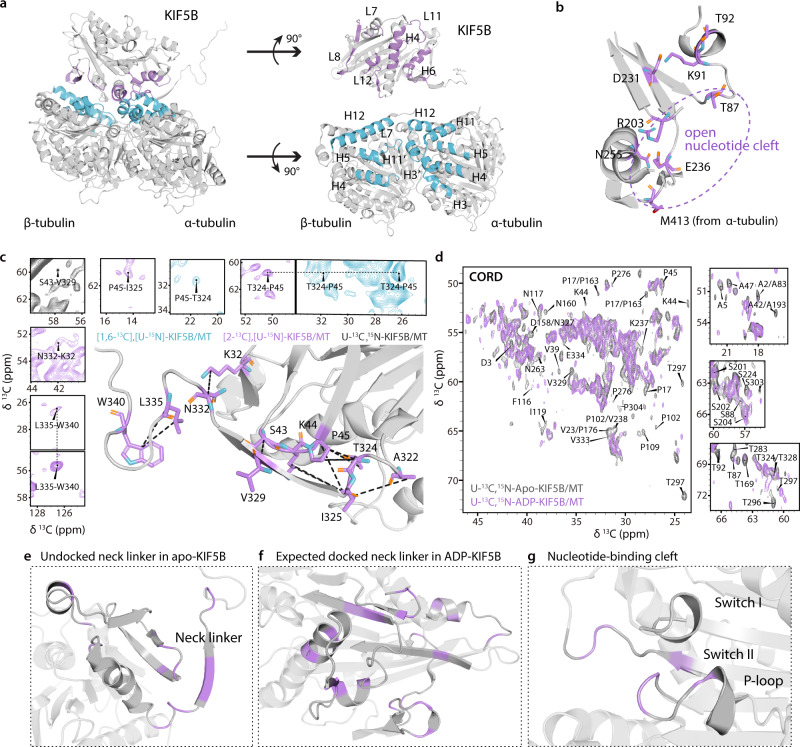
MAS NMR spectra and conformations of functionally important regions of KIF5B bound to MTs. **a** Left: binding site of KIF5B (purple) in complex with MTs (cyan); Right: An expanded view of the KIF5B-tubulin dimer interface showing the interacting helices and loops. **b** Nucleotide-binding region in the apo-KIF5B structure. The T87 in P-loop is distant from E236, indicating the “open” state of nucleotide-binding pocket. Carbon, nitrogen, oxygen and sulfur atoms are colored with purple, cyan, orange and red, respectively. Hydrogen atoms are not shown. **c** Expansions of 2D CORD spectra of [1,6-^13^C-glucose,U-^15^N]-KIF5B/MT (cyan), [2-^13^C-glucose,U-^15^N]-KIF5B/MT (purple) and U-^13^C,^15^N-KIF5B/MT (gray) illustrating long-range correlations with the neck linker. Distance restraint network between neck linker and nearby residues within KIF5B mapped onto the structure is shown in black dashed lines. **d** Expansion of the superposition of 2D CORD spectra of apo-KIF5B/MT (gray) and ADP-KIF5B/MT (purple) illustrating chemical shift and peak intensity changes. Assigned peaks with pronounced changes are labeled in the spectra. **e**–**g** Regions that comprise residues for which chemical shift and/or intensity changes are observed in the CORD spectra, mapped onto MAS NMR structure of KIF5B bound to MTs. Undocked neck linker in apo-KIF5B **e**; Expected region for docked neck linker in ADP-KIF5B **f**; and nucleotide binding cleft **g**.

The integrated structure of MAS KIF5B bound to MTs depicted in Fig. 3a provides important information about the binding interface: apo-KIF5B binds to both α- and β- tubulin in a single tubulin heterodimer. The main functional binding site resides on helix 4 (H4_k_) of KIF5B, which interacts with helix 11’ (H11’_α_) of α-tubulin. Within these two helical regions, the interaction between N255 on H4_k_ of KIF5B and M413 in H11’_α_ of α-tubulin (Fig. 3b) appears to be critical for the opening and closure of the nucleotide cleft. We identified multiple experimental distance restraints for N255 and the neighboring residues, from which the position and sidechain orientation of residue N255 could be defined. These restraints include N255C_β_-S235C_β_, A251C_β_-L248C_α_, A251C_β_-K237C_α_, A251C_α_-I254C_α_, K256C_α_-S289C_β_ and S257C_α_-T283C_α_. This is consistent with prior studies showing that the N255K mutation increases binding affinity of KIF5B to MTs in all nucleotide-bound states^34,49^. Other binding sites for α-tubulin and β-tubulin include loop 11 (L11_k_) and helix 6 (H6_k_) of apo-KIF5B as well as loop 7 (L7_k_), loop 8 (L8_k_), loop 12 (L12_k_) and the short β sheets within these loops. The MAS NMR structure of KIF5B bound to MTs contains extensive KIF5B-tubulin binding interfaces that are not observed in the X-ray structure of KIF5B bound to a tubulin dimer and a DARPin (PDB ID: 4HNA^32^). These interactions include L11_k_, and L7_k_ and L8_k_ of KIF5B with a portion of the H3, H4_α_ and H5_α_ helices from α- and β-tubulins. This result is not surprising because the engineered tubulin dimers/DARPin used for crystallography do not recapitulate the curvature inherent to MTs nor any of the inter-filament interactions. Thus, our current structure lays foundations for atomic-level understanding of the KIF5B-MT interactions.

### Conformations of nucleotide-binding motif and neck linker of apo-KIF5B bound to microtubules

Previous studies have shown details of the P-loop, comprising residues G85-T92, for nucleotide binding of KIF5B. T92 is the primary contact site for magnesium, while K91 and T87 coordinate the phosphate^32,34,50^. In ATP-KIF5B or ADP-KIF5B, T92 and D231 coordinate magnesium allowing for binding of K91 with the terminal phosphate while T87 binds to phosphate at the other end. In this manner the P-loop is brought close to Switch II region in ADP- and ATP-KIF5B (residues D231-V238) and positions the T87 and E236 side chains for interactions with the nucleotide-binding pocket in the “closed” state. In contrast, in the nucleotide-free apo-KIF5B state, D231 interacts with K91 instead of T92, preventing K91 from coordinating the phosphate. In our MAS NMR structure of apo-KIF5B, D231 also engages in an electrostatic contact with K91 with a distance of 3.8 Å between D231 C_γ_ and K91 C_ε_. As a result, T87 is more distant from E236, which is consistent with the “open” state of the nucleotide binding pocket (Fig. 3b). The position of Switch II segment is constrained by unambiguous experimental restraints involving residues adjacent to E236, such as K237C_α_-A251C_β_ and S235C_β_-Y84C_δ_. Furthermore, no correlation was detected between T87 and E236, indicating that these two residues are separated by more than 10 Å. Taken together, these results for nucleotide-free KIF5B are consistent with a previously determined cryo-EM structure of apo-KIF5B (PDB ID: 3J8X^34^), where the nucleotide pocket is in an “open” state (Supplementary Fig. 7a). Comparison of the MAS NMR-derived structure of apo-state KIF5B and the cryo-EM structure of ADP-AlF_4_^-^-KIF5B (PDB ID: 4HNA^32^) clearly reveals the “open” and “closed” state of the nucleotide pocket (Supplementary Fig. 7b). These above results will inform future studies into the structural switch of KIF5B during ADP binding and release.

Remarkably, the neck linker (residues K323-L335), for which electron density is missing in the cryo-EM and X-ray structures^23,24,34^, is well defined in the MAS NMR structure, as illustrated in Fig. 3c. Multiple strong cross peaks in the CORD spectra were unambiguously assigned and provide long-range restraints within the neck linker, including T324-P45, I325-K44, I325-P45, V329-S43, N332-K32 and L335-W340 (Fig. 3c). Several distance restraints between the neck linker and the adjacent segments were also identified. The position and orientation of the neck linker in the structure, calculated based on MAS NMR restraints, are consistent with the neck linker conformation observed experimentally by FRET and suggested by molecular dynamics simulations^23,24,34^, as shown in Supplementary Fig. 9. The residue-specific information about the neck linker conformation described above provides important experimental evidence that the neck linker is pointed in the opposite direction of the KIF5B movement. These findings validate the proposed model for the mechanism of kinesin motors walking along MTs^16^ and lay the foundations for understanding the role of the neck linker in the kinesin’s motility on microtubules.

### Structural and dynamic changes in ADP-KIF5B bound to MTs

Multiple chemical shift perturbations (CSPs) and attenuated peak intensities were observed in the spectra of U-^13^C,^15^N-ADP-KIF5B/MT, compared to apo-KIF5B/MT (Fig. 3d). These pronounced spectral differences indicate that the structure and/or dynamics is altered by binding of ADP to KIF5B. As shown in Fig. 3d-f, resonances of residues for which CSPs and/or intensity changes are seen are located in three regions of KIF5B. The first set of residues, T324, I325, N327, T328, V329, V333 and E334, belong to the neck linker and the proximal regions, encompassing P17, V23, V39, A42, K44, A47, T87, S88, T92, S303 and P304 (Fig. 3d). The second region spans residues A2, D3, A5, N117, I119, L126, D158, N160, P163, T169, P176, S224, N263, I265, V275, P276, M282, T283, L286, T296-I298, which are important for neck linker docking (Fig. 3e). The third region lies within the nucleotide-binding cleft and includes A233, K237 and V238 in Switch II as well as T87, S88 and K91 in the P-loop. Previous studies have shown that the ADP-KIF5B has a much weaker binding affinity for MTs than apo-KIF5B and ATP-KIF5B, and the neck linker of ADP-state kinesin is in equilibrium between the docked and undocked positions^19,21^. According to the proposed mechanism of kinesin’s movement along MTs, the ADP-KIF5B detaches from MT while the neck linker remains docked, the leading motor head moves toward the plus end as the neck linker becomes undocked, followed by the leading head’s binding to MT again, concomitant with ADP release^16,20^. Therefore, the ADP-KIF5B bound to MTs is expected to be in two states, with neck linker in either docked or undocked position. The observation of multiple residues exhibiting CSPs and peak intensity changes in the NMR spectra clearly indicates that a significant portion of ADP-KIF5B is in the state with neck linker docked, and not undocked as in apo-KIF5B (Supplementary Fig. 7c). Importantly, the changes in the regions listed above, especially for undocked and docked neck linkers, are compatible with kinesin’s walking on MTs.

Taken together, the MAS NMR results presented here provide valuable site-specific information that supporting previous biochemical studies and help clarify and solidify the binding mode of kinesin motor domains on MT filaments.

## Discussion

The mechanism of KIF5B walking on microtubules has been studied by biophysical methods, including single molecule trapping^14–16^. However, critical detailed structural features have been missing due to the lack of atomic-resolution structures of KIF5B bound to MTs. Several cryo-EM structures are available, with the resolution of ~ 5 Å^34^ or lower. At this resolution, important structural details cannot be discerned, such as the orientation of side chains that contact MTs. The most recent cryo-EM structure is at 3.6 Å resolution for GMPCPP-stabilized microtubules decorated with nucleotide-free kinesin^35^. Even at this resolution, the conformation of the functionally important neck linker and its position on the MTs could not be defined, most likely due to conformational flexibility in this region. MAS NMR spectroscopy was in a unique position to elucidate these details and informed on the structure, including the conformations of side chains as well as dynamic regions, such as the neck linker. Furthermore, since NMR observables are exquisitely sensitive to the local environments, differences between the apo state and several nucleotide-bound states were easily mapped.

The MAS NMR structure of KIF5B bound to MTs closed a number of important knowledge gaps and provided atomically detailed insights unavailable from other methods. One exciting finding is a well-defined undocked neck linker of apo-KIF5B. Specifically, the undocked neck linker is positioned near the β2 strand in all 22 subunits, providing experimental evidence for the hypothesized position of the undocked neck linker put forth on the basis of low-resolution FRET and MD simulation studies^23,24^. In addition, the MAS NMR results reported herein support the opening of the nucleotide-binding pocket, which is an important structural feature of nucleotide-free KIF5B, as observed in the earlier structures. Furthermore, all α-helical and β-strands delineated in the MAS-NMR structure of apo-KIF5B-MT assemblies are consistent with the secondary structure described for the cryo-EM structure (PDB ID: 3J8X) (Supplementary Fig. 5). The structure calculations using different structural states of KIF5B as initial models validate the final structure of single-chain KIF5B and corroborate the fact that the structures are driven by experimental MAS NMR restraints and not the starting coordinates. Specifically, irrespective of the starting models, in the final structures calculated with MAS NMR restraints, the nucleotide-binding pockets consistently adopt the “open” conformations, a characteristic of apo-KIF5B. Taken together, these results indicate that the kinesin structures were not trapped in a local minimum during the calculation.

Overall, the structure of nucleotide-free KIF5B bound to MTs represents an important advance, enabled by exciting recent developments in MAS NMR probe technology and progress in integrated methodological approaches. The novel CPMAS Cryoprobe made acquisition of high-quality 3D NCACX and NCOCX spectra possible, which in turn permitted unambiguous chemical shift assignments for 82.8% of KIF5B residues. By integrating MAS NMR restraints and the cryo-EM density map, several critical structural features were revealed for understanding the molecular mechanism of KIF5B’s processivity on MTs. The orientation of the neck linker and nucleotide-binding motifs determined in this study as well as the differences in conformational and dynamic profiles in apo- and ADP- states of KIF5B provide clues as to how local structural and dynamic changes translate into long-range motions. The intriguing observation that local dynamics of KIF5B differs in the nucleotide-bound and nucleotide-free states warrants further investigations into residue-specific motions of these key functional regions. Such future studies on both monomeric and dimeric KIF5B will provide a more thorough understanding how motions are coordinated or transmitted between the two motor domains. It will also be intriguing to probe intermolecular interactions between the two kinesin motor heads and between kinesin and microtubules. The surprising observation in the current study that the dynamic neck linker points opposite to the direction of kinesin’s movement opens up exciting possibilities to study conformational and dynamic changes of mobile regions in motor proteins that mediate directional movements. Looking forward, emerging MAS NMR technologies, such as ultrahigh magnetic fields (28.2 T) and ultrafast MAS probes capable of spinning frequencies of 100–150 kHz, delivering dramatic sensitivity and resolution enhancements^51–54^, will open doors for structural and dynamics studies of very large microtubule-based protein assemblies, with atomic level detail.

## Methods

### Materials

Common chemicals were purchased from Fisher Scientific and Sigma-Aldrich. Porcine brain tubulin, GTP and paclitaxel were purchased from Cytoskeleton, Inc. 99.8% D_2_O, ^15^NH_4_Cl and U-^13^C_6_ glucose, 2-^13^C glucose, 1,6-^13^C glucose, U-^13^C_6_,D_7_ glucose were purchased from Cambridge Laboratories, Inc. EDTA-free protease inhibitor tablets were obtained from Roche. Chromatography columns were purchased from GE Healthcare. The 400 mesh copper grids coated with formvar and stabilized with evaporated carbon films were purchased from Electron Microscopy Science.

The human KIF5B motor domain construct (amino acids 1–349) was prepared using the pET28b vector fused with a His_6_-SMT3 Tag at the N-terminus (in the form of His_6_-SMT3-KIF5B). The His_6_-SMT3-KIF5B was transformed into *Escherichia coli* BL21(DE3) cells. The His_6_-Ulp1 was expressed and purified from the His_6_-Ulp1 protease construct kindly provided by Dr. Christopher Lima from HHMI and Memorial Sloan Kettering Cancer Center, New York, NY 10065.

### Expression and purification of KIF5B

The U-^13^C, ^15^N-enriched KIF5B was expressed in *E.coli* cells following a protocol developed earlier with modifications^55^. Cells were grown in 20 mL Luria-Bertani (LB) media containing 30 mg/L kanamycin for 6–8 h at 37 °C until the OD reached 1.2–1.6. The cell pellets obtained by centrifugation at 4000 g for 5 min were resuspended into 50 mL M9 minimal media and grown at 37 °C overnight. The overnight culture was transferred into 1 L M9 minimal media (supplemented with 1 mM ZnSO_4_, 0.1 mM CaCl_2_, 1 mg/L Biotin, 0.5 mg/L Thiamin, 30 mg/L kanamycin, 2 g/L ^15^NH_4_Cl and 4 g/L U-^13^C_6_ glucose) to an OD of 0.1–0.2. Cells were grown at 37 °C until the OD reached 0.6–0.8, induced with 0.8 mM IPTG, and the protein was expressed for 4 hr at 37 °C. Cells were harvested by centrifugation at 4000 g for 30 min at 4 °C, resuspended in 40 mL of phosphate buffered saline (PBS) buffer (10 mM Na_2_HPO_4_, 2 mM KH_2_PO_4_, 137 mM NaCl, 2.7 mM KCl, 1 mM DTT, pH 7.4) and were stored at −80 °C if were not directly used for protein purification.

Sparsely-^13^C-enriched and U-^15^N-enriched KIF5B was expressed following the same expression protocol as described above with the carbon source substitution of 2-^13^C glucose or 1,6-^13^C glucose for U-^13^C_6_ glucose.

U-^2^H, ^13^C, ^15^N-enriched KIF5B was expressed in M9 minimal media prepared in 99.8% D_2_O and supplemented with U-^13^C_6_,D_7_ glucose and ^15^NH_4_Cl. Cells were grown in 20 mL LB media containing 30 mg/L kanamycin overnight at 37 °C, pelleted down and resuspended in 50 mL 100% H_2_O M9 minimal media (supplemented with 1 mM ZnSO_4_, 0.1 mM CaCl_2_, 1 mg/L Biotin, 0.5 mg/L Thiamin, 30 mg/L kanamycin, 2 g/L ^15^NH_4_Cl and 4 g/L ^12^C,^1^H glucose). After growth at 37 °C to an OD of ~1.5, cells were centrifuged at 4000 g for 20 min, resuspended in 75 mL 70% D_2_O M9 minimal media containing 2 g/L ^15^NH_4_Cl and 4 g/L ^12^C,^1^H glucose and grown at 37 °C to an OD of ~ 3. Cells were harvested at 4000 g for 20 min and resuspended in 150 mL 99.8% D_2_O M9 minimal media (supplemented with 1 mM ZnSO_4_, 0.1 mM CaCl_2_, 1 mg/L Biotin, 0.5 mg/L Thiamin, 30 mg/L kanamycin, 2 g/L ^15^NH_4_Cl and 4 g/L ^12^C,^1^H glucose). After an overnight growth, cells were centrifuged at 4000 g for 20 min and resuspended in 1 L 99.8% D_2_O M9 minimal media containing 2 g ^15^NH_4_Cl and 4 g U-^13^C_6_,D_7_ glucose as the sole nitrogen and carbon sources. The resulting culture was grown at 37 °C until the OD reached 0.6–0.8, induced with 0.8 mM IPTG, and grown at 37 °C for 4 h for protein expression. Cells were harvested by centrifugation at 4000 g for 30 min at 4 °C, resuspended in 40 mL of phosphate buffered saline (PBS) buffer and were stored at −80 °C or directly used for protein purification.

U-^13^C,^15^N-enriched KIF5B, sparsely-^13^C-enriched and U-^15^N-enriched KIF5B and U-^2^H, ^13^C, ^15^N-enriched KIF5B were all purified by the same two-step affinity chromatography. Cells containing the overexpressed protein were thawed thoroughly on ice, followed by the addition of one tablet of EDTA-free protease inhibitor and 200 mM DTT to a final concentration of 1 mM. The cells were then sonicated with 38% amplitude for 20 min (pulse on and off time  of 10 s) in an ice bath. Cell debris was removed by centrifugation at 27,000 g for 30 min at 4 °C. The supernatant was filtered through a 0.2 mm filter and loaded onto a HisTrap Ni-affinity column (5 mL, GE Healthcare). The target protein was eluted with a 120–240 mM imidazole gradient in PBS buffer. The His_6_-SMT3-fused U-^13^C,^15^N-enriched KIF5B was treated with the His_6_-Ulp1 protease at 4 °C overnight to remove the His_6_-SMT3tag. The resulting solution was loaded onto a HisTrap Ni-affinity column (5 mL, GE Healthcare) and was eluted with 80 mM imidazole in PBS buffer. Fractions containing the target protein were combined and exchanged into 25 mM PIPES buffer (1 mM EGTA, 1 mM MgCl_2_, 1 mM DTT, 100 mM KCl, pH 6.8) The resulting solution of pure KIF5B was concentrated to 8–10 mg/mL for further preparation of KIF5B/MT complexes.

### Preparation of paclitaxel-stabilized MTs

Porcine brain tubulins were dissolved in 80 mM PIPES buffer (1 mM MgCl_2_, 1 mM EGTA, pH 6.8), and the resulting solution was pre-cleared by ultracentrifugation at 435,400 g at 4 °C for 10 min. The tubulin supernatant was incubated with 20–25 μM paclitaxel and an equal volume of 80 mM PIPES buffer (supplemented with 2 mM GTP, 20% (v/v) DMSO) for 30 min at 37 °C. The polymerized MTs were ultracentrifuged at 108,900 g at 20 °C for 10 min.

### Preparation of KIF5B/MT complexes for MAS NMR spectroscopy

KIF5B/MT complexes were prepared following a protocol previously developed for the CAP-Gly/MT complexes with modifications to obtain NMR samples that have high stability and homogeneity^44^. Specifically, the concentrated KIF5B solution was pre-cleared at 435,400 g at 4 °C for 10 min before use. To assess the binding of KIF5B to paclitaxel-stabilized MTs, fresh polymerized MTs were gently resuspended into the concentrated KIF5B with 20–25 μM paclitaxel to a 2:1 optimal molar ratio of KIF5B:tubulin dimer. The mixture was incubated at 25 °C for 30 min. The resulting complex was pelleted by ultracentrifugation at 156,800 g at 20 °C for 30–60 min. For U-^13^C,^15^N-enriched KIF5B/MT complexes, ADP stock solution (40 mM) was added to KIF5B stock solution to reach a final concentration of 1 mM ADP and was stored on ice at 4 °C for overnight before forming the ADP-KIF5B/MT complexes. For SDS-PAGE analysis, 20 μL of the supernatant and the pellets suspension were mixed with same volume of 2X SDS sample buffer; 10 μL of the mixture was loaded onto 15% acrylamide gels. Supplementary Fig. 1 shows the expression for KIF5B and the binding assays KIF5B/MT performed under various conditions. Unprocessed gel images are provided in the Source Data.

For MAS NMR experiments using a Bruker EFree 3.2 mm HCN probe, the hydrated pellets of [U-^13^C,^15^N]-enriched KIF5B/MT complexes (total weight of 54.5 mg, corresponding to ca. 5 mg of [U-^13^C,^15^N]-KIF5B) and [1,6-^13^C-glucose,U-^15^N]-enriched KIF5B/MT complexes (total weight of 55 mg, containing ca. 4.5 mg of [1,6-^13^C-glucose,U-^15^N]-KIF5B) were transferred into two thin-wall 3.2 mm rotors. For MAS NMR experiments with the CPMAS CryoProbe, the hydrated pellet of [U-^13^C,^15^N]-enriched KIF5B/MT complexes (82.4 mg total weight, corresponding to ~ 7.5 mg of [U-^13^C,^15^N]-KIF5B) and [2-^13^C-glucose,U-^15^N]-enriched KIF5B/MT complexes (55 mg total weight, corresponding to ~ 4.2 mg of [2-^13^C-glucose,U-^15^N]-KIF5B) were transferred into two thin-wall 3.2 mm Bruker CPMAS CryoProbe rotors. For MAS NMR experiments using a Bruker 1.9 mm HCN probe, the hydrated pellets of [U-^2^H,^13^C,^15^N]-enriched KIF5B/MT complexes (10 mg in total, corresponding to ~ 1.1 mg of [U-^2^H,^13^C,^15^N]-KIF5B), [U-^13^C,^15^N]-enriched KIF5B/MT complexes (total weight of 16.0 mg, corresponding to ~ 1.9 mg of [U-^13^C,^15^N]-KIF5B), and [U-^13^C,^15^N]-enriched ADP-KIF5B/MT complexes (16.1 mg total weight, corresponding to ~1.8 mg of [U-^13^C,^15^N]-ADP-KIF5B) were transferred into three 1.9 mm MAS rotors. For MAS NMR experiments using a Bruker 1.3 mm HCN probe, the hydrated pellet of [U-^13^C,^15^N]-enriched KIF5B/MT complexes (4.4 mg total weight, corresponding to ~ 0.6 mg of [U-^13^C,^15^N]-KIF5B) was transferred into a 1.3 mm rotor.

### Transmission electron microscopy (TEM)

The morphology of KIF5B/MT complexes was characterized by transmission electron microscopy (TEM). Samples of 5–10 μM complexes were stained with uranyl acetate (5% w/v), deposited onto 400 mesh, formvar/carbon-coated copper grids and air-dried for 40–60 min. TEM analysis was performed with a Zeiss Libra 120 transmission electron microscope operating at 120 kV.

### MAS NMR spectroscopy

MAS NMR spectra of [U-^13^C,^15^N]-KIF5B/MT, [1,6-^13^C-glucose,U,^15^N]-enriched KIF5B/MT, and [U-^13^C,^15^N]-ADP-KIF5B/MT were acquired on a Bruker 20.0 T narrow bore AVIII spectrometer outfitted with a 3.2 mm E-Free HCN probe. The Larmor frequencies were 850.4 MHz for ^1^H, 213.8 MHz for ^13^C, and 86.2 MHz for ^15^N. The MAS frequency was 14 kHz maintained at ± 10 Hz by a Bruker MAS controller. The temperature was calibrated with KBr^56^ and the actual sample temperature was maintained at 10.0 ± 0.5 °C using a Bruker BCUII temperature controller for all acquisitions. ^13^C and ^15^N chemical shifts were referenced with respect to external standards adamantine and ammonium chloride, respectively^57^. Typical 90° pulse lengths were 2.7 μs for ^1^H, 2.9 μs for ^13^C, and 4.7 for ^15^N. The ^1^H -^13^C and ^1^H -^15^N cross polarization (CP) were performed with a linear amplitude ramp of 90–110% at the ^1^H radio frequency (rf) field of 93 kHz; the center of the ramp on the ^13^C or ^15^N was matched to the first spinning sideband of the Hartmann-Hahn condition. The contact times for ^1^H -^13^C and ^1^H -^15^N CP steps were 1.0 ms and 1.2 ms, respectively. Typical radio frequency (rf) fields for double cross polarization (DCP) were 45 kHz on ^13^C and 30 kHz on ^15^N with optimized ^15^N-^13^C DCP contact time of 4.5 ms with a tangent amplitude ramp (90%–110%). SPINAL-64 decoupling^58^ was applied at a rf field strength of 80 kHz during the evolution and acquisition periods.

MAS NMR spectra of [U-^13^C,^15^N]-KIF5B/MT complexes were also acquired on a Bruker 14.1 T narrow bore Neo spectrometer outfitted with a 3.2 mm CPMAS CryoProbe. The Larmor frequencies were 600.3 MHz for ^1^H, 150.9 MHz for ^13^C, and 60.8 MHz for ^15^N. The MAS frequency was 14.000 ± 0.002 kHz by a Bruker MAS controller. The temperature was calibrated with KBr^56^ and the sample temperature was maintained at 10 ± 1 °C. The typical 90° pulse lengths were 3.1 μs for ^1^H, 4.0 μs for ^13^C, and 5.5 μs for ^15^N. The ^1^H -^13^C and ^1^H -^15^N cross polarization (CP) were performed with a 30% linear amplitude ramp on ^1^H, with the ^13^C/^15^N rf field of 55/45 kHz matched to Hartmann-Hahn conditions at the first spinning sideband. The contact times for ^1^H-^13^C and ^1^H-^15^N CP steps were 1.0 ms and 1.2 ms, respectively. Typical DCP rf fields of 18.3–18.7 kHz on ^13^C and 4.7 kHz on ^15^N were used with a ^15^N-^13^C DCP contact time of 6 ms. A 30 and 25% tangential amplitude ramp was applied on ^13^C with the rf carrier frequencies placed at 190 and 35 ppm in NCO and NCA transfers, respectively. TPPM decoupling^59^ was applied at a rf field strength of 80 kHz during the evolution and acquisition periods.

Proton-detected 2D NH HETCOR MAS NMR spectra of [U-^13^C,^15^N]-KIF5B/MT and [U-^2^H,^13^C,^15^N]-KIF5B/MT complexes were acquired on a Bruker 20.0 T narrow bore AVIII spectrometer outfitted with a 1.3 mm HCN probe. The Larmor frequencies were 850.4 MHz for ^1^H, 213.8 MHz for ^13^C, and 86.2 MHz for ^15^N. The MAS frequency was 60 kHz maintained at ±10 Hz by a Bruker MAS controller. The temperature was calibrated with KBr^56^ and the sample temperature was maintained at 17.0 ± 0.3 °C. ^1^H and ^15^N chemical shifts were referenced with respect to external standards adamantine and ammonium chloride, respectively^57^. Typical 90° pulse lengths were 1.72–3.3 μs for ^1^H. The ^1^H-^15^N CP was performed with a linear ramp of 90–110% at the ^1^H radio frequency (rf) field of 156 kHz; the center of the ramp on the ^15^N was 99 kHz and matched to the first spinning sideband of the Hartmann-Hahn condition. Low-power saturation was applied at a rf field of 5 kHz for water signal suppression. ^1^H decoupling power of 15 kHz was used during acquisition.

### NMR data processing and analysis

All MAS NMR spectra were processed in either Bruker TopSpin or NMRPipe^60^ and were analyzed using Sparky^61^ and CcpNmr Analysis version 2^62^. All the 2D and 3D spectra were processed with 90°, 60° or 45° shifted sine bell apodization followed by a Lorentzian-to Gaussian transformation in both dimensions. For spectra acquired with Bruker 3.2 mm EFree HCN probe, forward linear prediction to twice the number of the original data points was used in the indirect dimension, followed by zero filling to twice the total number of points. Detailed processing parameters are shown in Supplementary Table 1.

### Structure calculation of KIF5B bound to MTs

#### Energy minimization of a single subunit

Secondary structure elements of KIF5B bound to polymerized MTs were predicted by IDP/IUP^63–66^, CSI3.0^67^ and TALOS-N^62^ from the NMR chemical shifts. Unambiguous and ambiguous Xplor distance restraints were generated from assigned cross-peaks in experimental CORD spectra. A total of 1339 distance restraints and 494 backbone torsion angle restraints were utilized in structure calculation using Xplor-NIH (version 2.53)^46–48^. Ambiguous restraints exceeding 5-fold ambiguity were not considered. Bounds of the distance restraints were set to 1.5–6.5 Å (4.0 ± 2.5 Å) and 2.0–7.2 Å (4.6 ± 2.6 Å) for intra- and inter-residue restraints, respectively, consistent with our previous study^39^.

Standard terms for bond lengths, bond angles and improper angles were used to enforce correct covalent geometry. A statistical torsion angle potential^68^ and the gyration volume term were employed^69^. A hydrogen bond database term, HBPot, was used to improve hydrogen bond geometries^70^.

Xplor-NIH structure calculations for the single unit were seeded from the coordinates of the cryo-EM structure (PDB 3J8X^34^) and 100 structures were annealed. Missing residues were built with the protocol.addUnknownAtoms routine in Xplor-NIH. Two rounds of annealing were performed in the run at 3,000 K for 10 ps or 10,000 steps, whichever completed first. The starting time step was 1 fs and was self-adjusted in subsequent steps to ensure conservation of energy. The initial velocities were randomized about a Maxwell distribution using the starting temperature of 3000 K. Subsequently the temperatures were reduced to 25 K in steps of 25 K. At each temperature, the initial time step was set to the default value of 1 fs, and a 0.4-ps dynamics run was performed. Force constants for distance restraints were ramped from 2 to 30 kcal mol^−1^ Å^−2^. The dihedral restraint force constants were set to 10 kcal mol^−1^ rad^−2^ for high-temperature dynamics at 3000 K and 200 kcal mol^−1^ rad^−2^ during cooling.

A global envelope in the form of synthetic density with 8 Å resolution was prepared with UCSF Chimera^71^ to preserve the overall shape of the system using the coordinates of the cryo-EM structure (PDB 3J8X). The resulting map was implemented into the Xplor-NIH run with the cryo-EM potential to generate a cross-correlation probability distribution potential^38^. The potential was restricted to backbone (N, C′, Cα, O) atoms such to not distribute the sidechain orientations defined by NMR distance and dihedral restraints. The cryo-EM potential was only applied to residues that are present in this work and the cryoEM structure (PDB 3J8X) (residues 3–6, 8–167, 169–173, 175–320) and not those that are sequence mismatches from the starting coordinates. The force constant of the cross-correlation probability distribution potential was set to 50 kcal mol^−1^ during high-temperature dynamics and cooling. The gyration volume force constant was turned off to avoid conflicts with the cross-correlation potential. The annealed structures were minimized using a Powell energy minimization scheme in Cartesian space. The lowest-energy structure from the run was selected for the next step.

#### Docking into cryo-electron microscopy density

The lowest energy structure from the run described in the last subsection was subjected to rigid-body docking about the experimental cryo-EM density map (EMD-6187, PDB 3J8X, 6 Å resolution) using an in-house UCSF Chimera script. The protocol bears similarities to previous work from our laboratory^39^ with an important adaptation: instead of employing docking to identify the best fitting structure amongst many candidates in the cryo-EM density, the script identifies the best docking positions of a single structure. Here, 22 positions were identified about the map on the basis of the lowest cross-correlation values and brief visual inspection.

#### Joint refinement with cryo-EM density

After the docking we performed a joint refinement in Xplor-NIH using the 22 molecules and the experimental cryo-EM density (EMD-6187, PDB 3J8X) following the same protocol, parameters, and force constants as in the previous step. Each of the 22 molecules was assigned to a unique segment id (A-V). The protein structure file (PSF) of a single unit was loaded from the sequence file and expanded to all of the 22 subunits with the psfGen.duplicateSegment function in Xplor-NIH. Coordinates were loaded from 22 files with the initCoords protocol in Xplor-NIH. The experimental distance and dihedral restraints from the first Xplor-NIH structure calculation were applied to the 22 subunits with a loop in the python infrastructure of Xplor-NIH.

Approximate non-crystallographic symmetry was imposed using Xplor-NIH’s PosDiffPot term, allowing the 22 subunits to differ by up to 1 Å. The experimental cryo-EM map (EMD 6187, PDB 3J8X) was incorporated into the Xplor-NIH run with the cryo-EM potential to generate a cross-correlation probability distribution potential^38^. The calculation force constants were set to the same values as the previous step. As with the previous section, the annealed structures were minimized using a Powell energy minimization scheme in Cartesian space. The lowest-energy structure comprising 22 subunits from the run was selected for the next step.

#### Neck linker refinement

We performed a final round of Xplor-NIH structure calculations to incorporate 17 additional restraints for the neck linker region. For each of the 22 subunits we performed an individual run where 1000 structures were calculated. We followed the identical protocol, parameters, and force constants as during the first step refinement, except for one modification: to preserve the joint refinement with cryo-EM density only neck-linker and terminal residues (321–349) were permitted to move freely during dynamics, the remaining residues (1–320) were set to a rigid body. As with the previous two sections, the annealed structures underwent a Powell energy minimization in Cartesian space. The lowest energy structure from each of the runs, corresponding to each of the 22 subunits, was selected for the final ensemble. Lastly, each member of the final ensemble was returned to their initial fitting in the density after joint-refinement by aligning those residues that are not in the neck-linker or terminus (1–320) followed by a local density fitting in UCSF Chimera^71^. A flowchart of the structure determination protocol for KIF5B bound to MTs is shown in Supplementary Fig. 8.

#### Structure validation

To validate the MAS NMR structure of apo-KIF5B, we performed structure calculations of KIF5B using the structures of kinesin’s different nucleotide states as the initial models. The X-ray structure of ADP-KIF5B (PDB 4HNA) and cryo-EM structure of ATP-KIF5B (PDB 3J8Y) were used as the starting coordinates; the structures were calculated and refined using the MAS NMR restraints of apo-KIF5B bound to MTs determined in this work. The structures calculated following this protocol are different from the starting coordinates and converge to the same NMR structure calculated from the initial model of apo-KIF5B (PDB 3J8X). These validations ascertain that the structures are defined by experimental MAS NMR restraints.

### Structure analysis and visualization

Atomic R.M.S. differences were calculated using routines in Xplor-NIH (version 2.53)^46–48^. The structural ensembles were rendered for visualization in PyMOL using in-house shell/bash scripts. Renders of structures docked into the cryo-EM density were performed with UCSF Chimera^71^ with in-house UCSF Chimera python scripts. Secondary structure elements were classified according to TALOS-N and manual inspection.

### Reporting summary

Further information on research design is available in the Nature Research Reporting Summary linked to this article.

## Supplementary information

Supplementary information

Reporting Summary

## Data Availability

The coordinates for the all atom NMR structures of apo-KIF5B bound to MTs have been deposited in the Protein Data Bank under accession codes PDB 7RIK. The MAS NMR chemical shifts have been deposited in the Biological Magnetic Resonance Data Bank under accession code 30936. The Cryo-EM density used in this study are available in the EM data bank and Protein Data Bank under accession codes EMD-6187 and PDB 3J8X. Source data are provided in this paper.

## Source data

Source Data
