# Supplementary material for: Magic-angle-spinning NMR structure of the kinesin-1 motor domain assembled with microtubules reveals the elusive neck linker orientation
Source: Nat Commun. 2022 Nov 10;13:6795. doi: 10.1038/s41467-022-34026-w (PMC9649657; doi:10.1038/s41467-022-34026-w)
Supplement: Supplementary file 1 — Supplementary information [file 41467_2022_34026_MOESM1_ESM.pdf]

Supplementary Information for

Magic-Angle-Spinning NMR Structure of the Kinesin-1 Motor Domain Assembled with Microtubules Reveals the Elusive Neck Linker Orientation

Chunting Zhang[1,#], Changmiao Guo[1,#], Ryan W. Russell[1], Caitlin M. Quinn[1], Mingyue Li[1], John C. Williams[3,*], Angela M. Gronenborn[2,*], and Tatyana Polenova[1,*]

[1]Department of Chemistry and Biochemistry, University of Delaware, Newark, DE 19716, United States; [2]Department of Structural Biology, University of Pittsburgh School of Medicine, 3501 Fifth Ave., Pittsburgh, PA 15261, United States; [3]Department of Molecular Medicine, Beckman Research Institute of City of Hope, 1500 East Duarte Road, Duarte, CA 91010, United States

***Corresponding authors:** Tatyana Polenova, Department of Chemistry and Biochemistry, University of Delaware, Newark, DE, USA, Email: tpolenov@udel.edu; Angela M. Gronenborn, Department of Structural Biology, University of Pittsburgh School of Medicine, 3501 Fifth Ave., Pittsburgh, PA 15260, USA, Email: amg100@pitt.edu; John C. Williams, Department of Molecular Medicine, Beckman Research Institute of City of Hope, 1500 East Duarte Road, Duarte, CA 91010, USA, Email: jcwilliams@coh.org

[#]These authors contributed equally

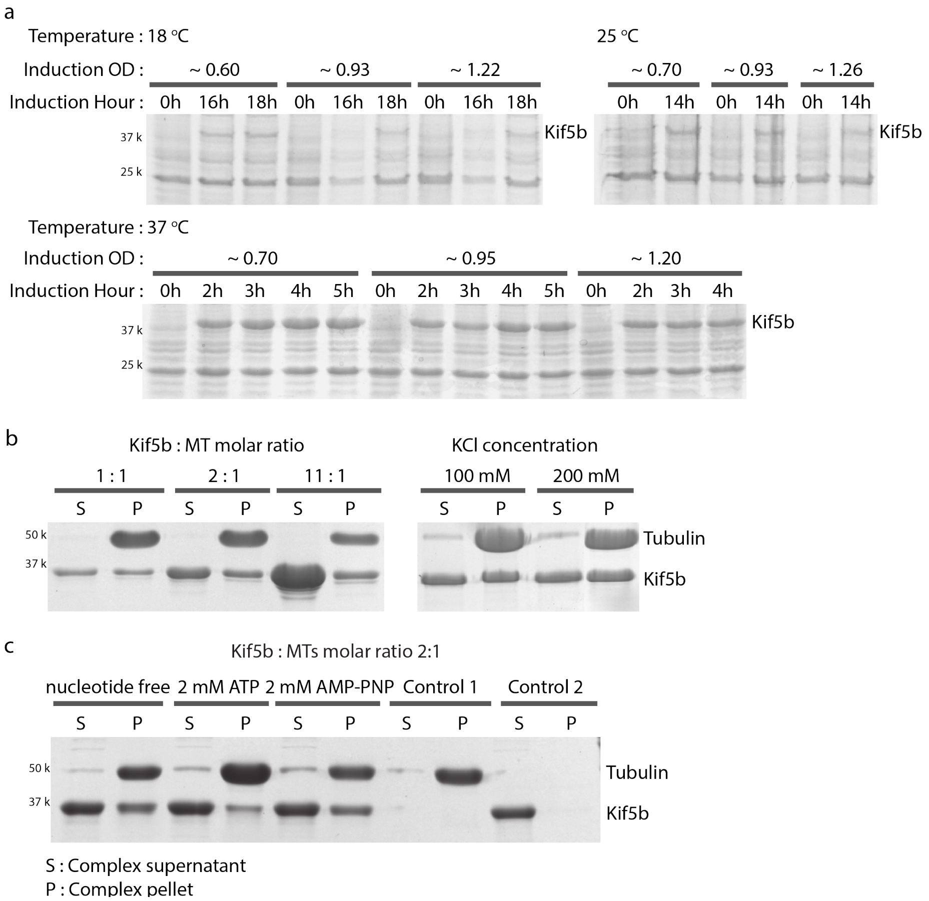

Supplementary Fig. 1. a SDS-PAGE analysis of the time course of KIF5B expression at 18 °C, 25 °C and 37 °C. To prepare the NMR samples, KIF5B was expressed at 37 °C for 4h with induction at OD ~0.7. **b** Co-sedimentation assays for assessment of KIF5B/MT complex formation. The typical KIF5B:MT molar ratio used for NMR sample preparation was 2:1 and samples were stored in BRB80 buffer (200 mM KCl) before packing into a MAS NMR rotor. The detailed KIF5B/MT complex preparation protocol is described in Materials and Methods. **c** Co-sedimentation assays of KIF5B/MT complex in the presence of different nucleotides. The experiments in **a**-**c** was replicated three times with similar results. Source data are provided as a Source Data file.

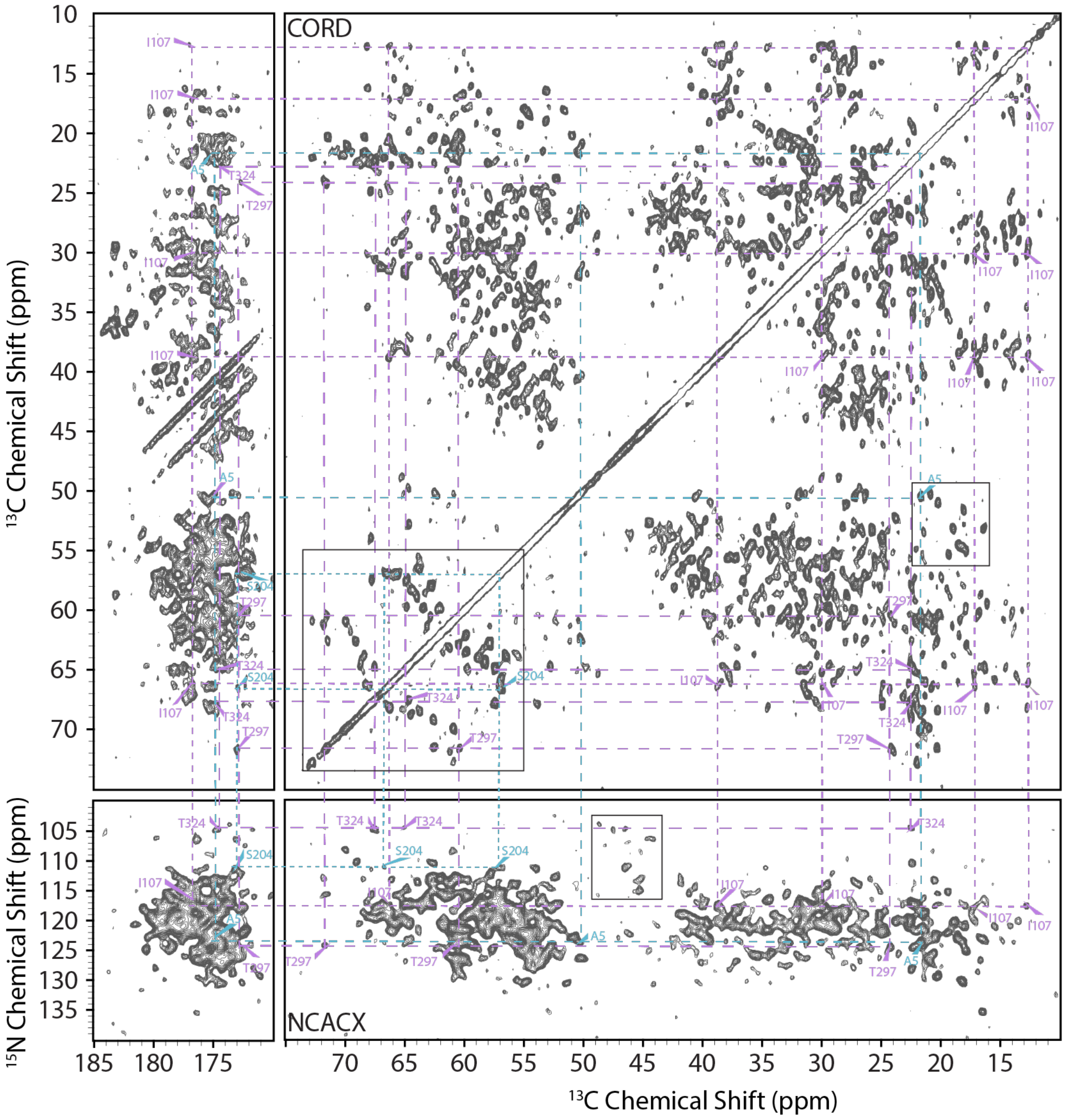

Supplementary Fig. 2. 2D CORD (top) and NCACX (bottom) spectra. Representative chemical shift assignments and internuclear correlations are labeled in the spectra. Walks through representative spin systems are shown for S204 (cyan) and T234 (magenta).

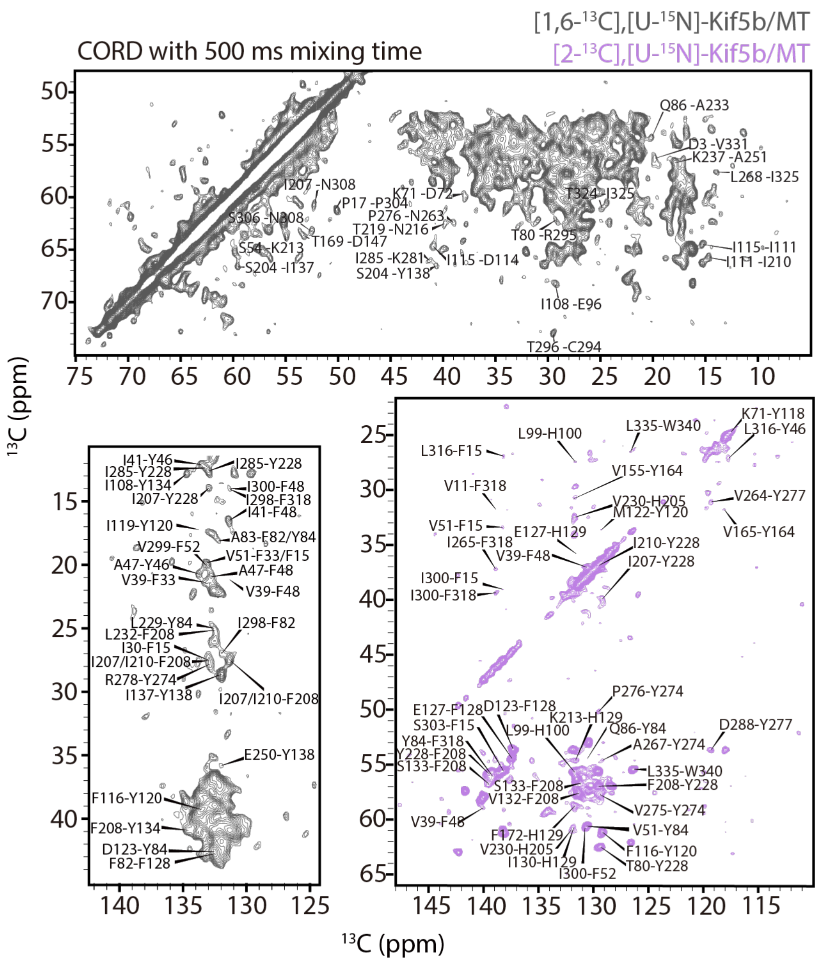

Supplementary Fig. 3. Expansions of 2D CORD spectra of [1,6-^{13}C]-[U-^{15}N]- KIF5B/MT (gray) and [2-^{13}C]-[U-^{15}N]- KIF5B/MT (purple) for 500 ms mixing times. Inter-residue correlations are labeled with amino acid type and number.

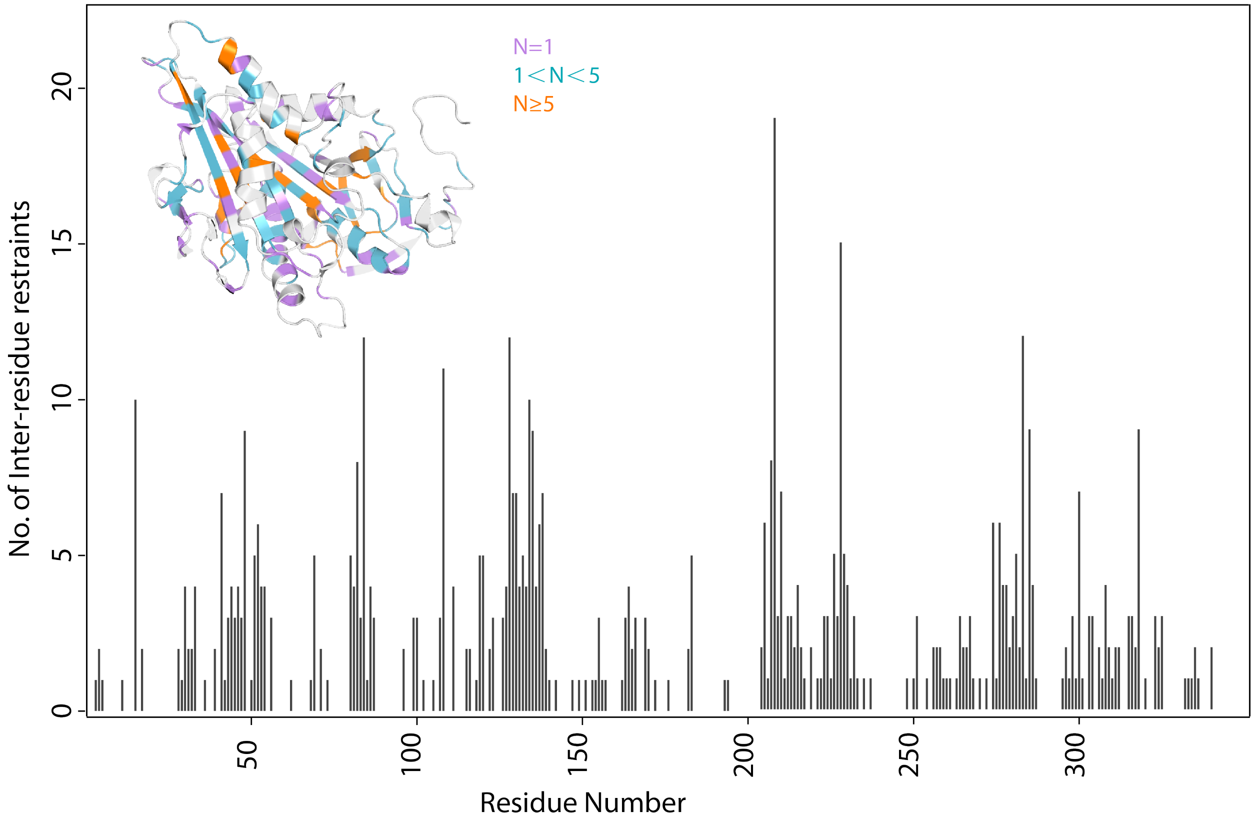

Supplementary Fig. 4. Number of inter-residue restraints per residue along the sequence of KIF5B. Inter-residue restraints per residue were mapped onto the MAS NMR structure of KIF5B, color-coded by number of restraints (N): purple, N=1; cyan, 1<N<5; orange, N≥5.

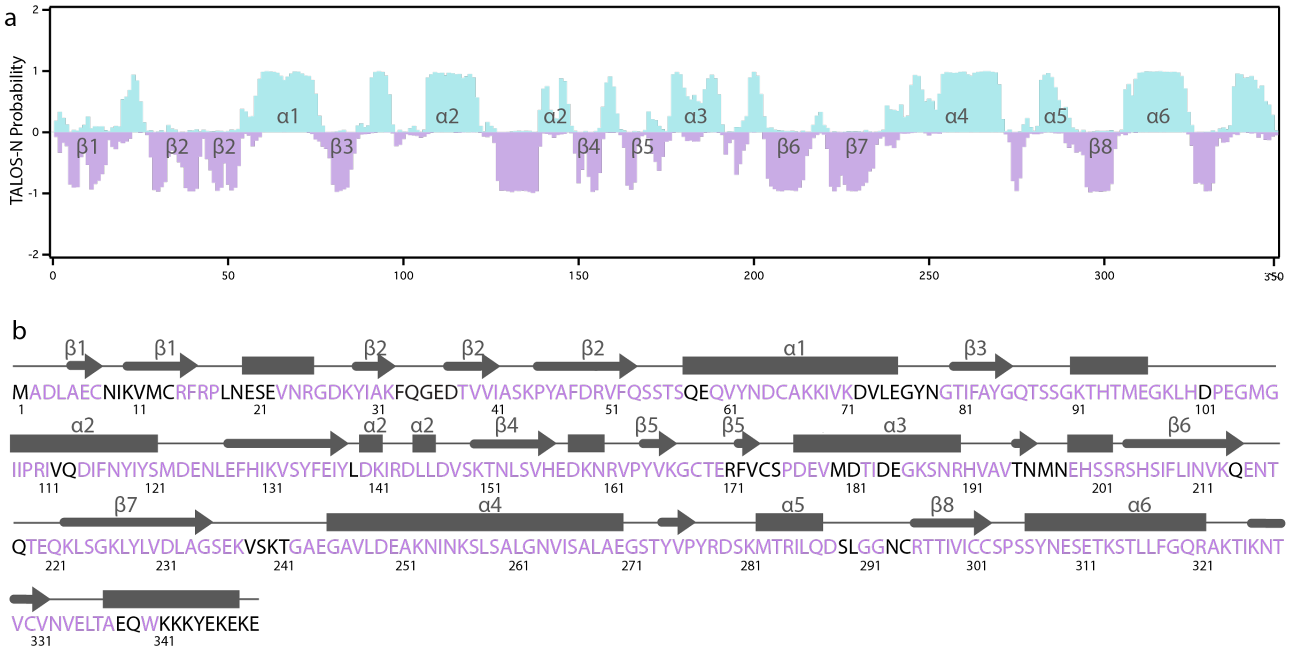

Supplementary Fig. 5. **Secondary structure analysis of KIF5B bound to MTs. a** Secondary structure prediction by TALOS-N, probability of α-helices (cyan) and β-strands (purple) for all residues, plotted against residue numbers. **b** Primary sequence and predicted secondary structure of KIF5B by TALOS-N. Secondary structure predicted for assigned residues are chemical shift based and for unassigned residues are sequence based. The arrows and rectangles represent β-strands and α-helices, respectively. Assigned residues are colored in purple.

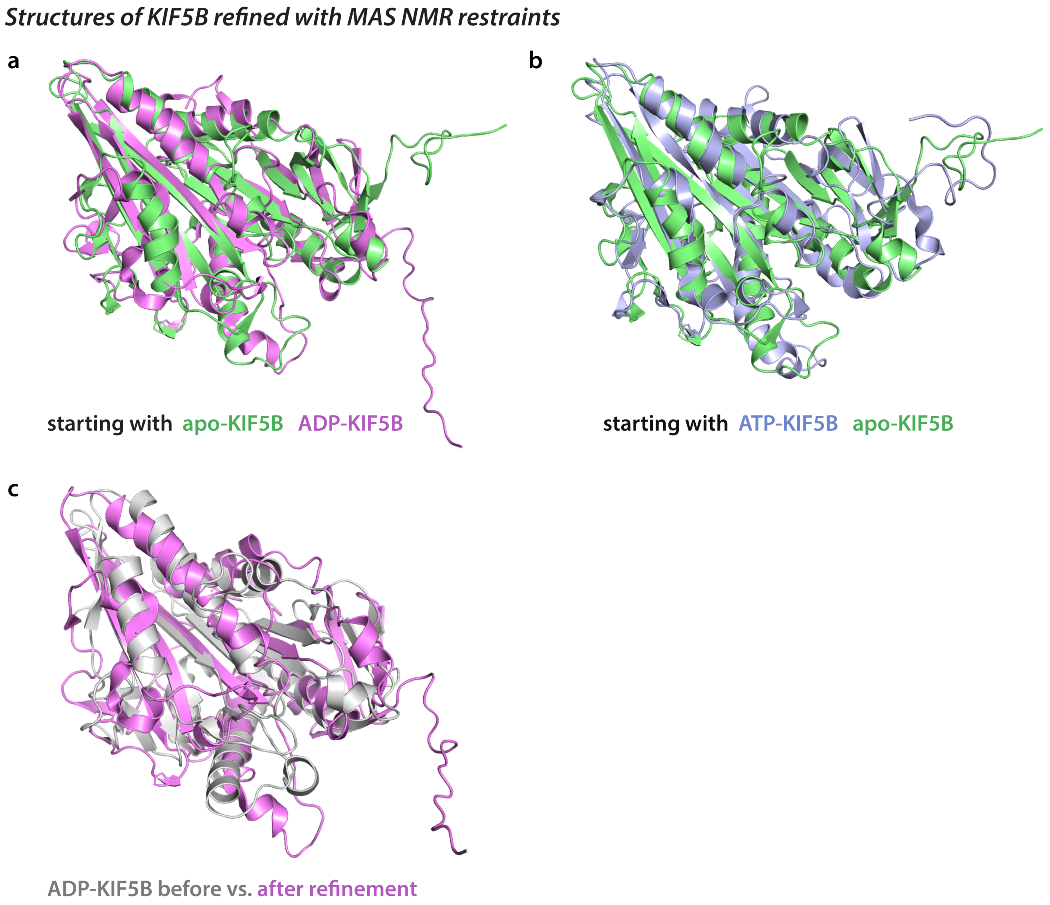

Supplementary Fig. 6. Structure validations of apo-KIF5B single chain using different nucleotide states of kinesin-1 as the initial models. **a,b** Structures of KIF5B motor domain that were calculated and refined with experimental MAS NMR restraints, starting with apo-state (green), ADP-state (magenta) and ATP-state (light blue) kinesin coordinates. The X-ray structure of ADP-KIF5B (PDB 4HNA) and cryo-EM structures of ATP-KIF5B (PDB 3J8Y) and apo-KIF5B (PDB 3J8X) were used as the initial models. **c** Structure comparison of ADP-KIF5B (PDB 4HNA) before (gray) and after (magenta) the refinements with NMR restraints. The resulting structures are the same as our structure calculated from the initial model of nucleotide-free KIF5B and different from the starting coordinates.

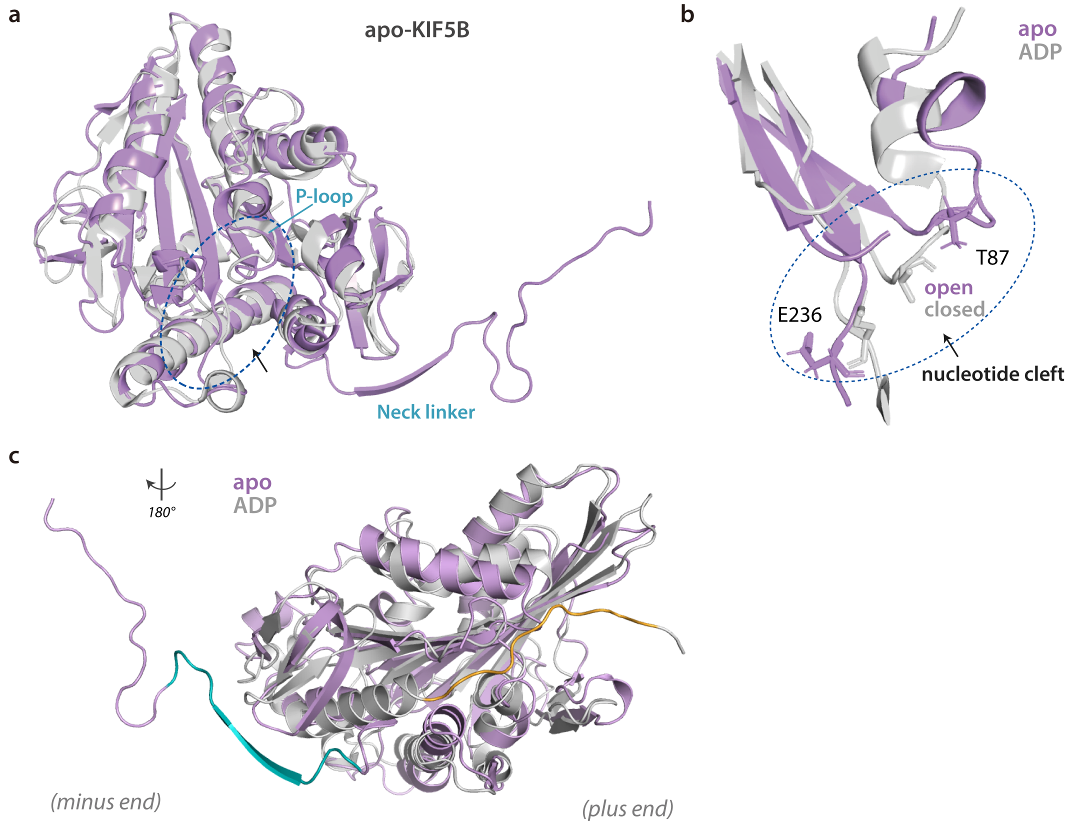

Supplementary Fig. 7. **a** Superposition of the MAS NMR-derived structure (purple) and cryo-EM structure (gray, PDB ID: 3J8X) of apo-KIF5B bound to paclitaxel-stabilized MTs. **b** Conformations of the nucleotide-binding region and neck linker in the MAS NMR structure of apo-KIF5B (purple) in comparison to those in the X-ray structure of ADP-KIF5B (gray, PDB ID: 4HNA). The nucleotide pocket adopts open and closed state in the nucleotide-free and ADP-bound state, respectively. The catalytic site E236 is distant from P-loop T87 in the open state (purple sticks), whereas E236 is proximal to T87 in the closed state (gray sticks). The neck linker is in the undocked position in apo-KIF5B, whereas a significant portion of ADP-KIF5B is in the state with neck linker docked.

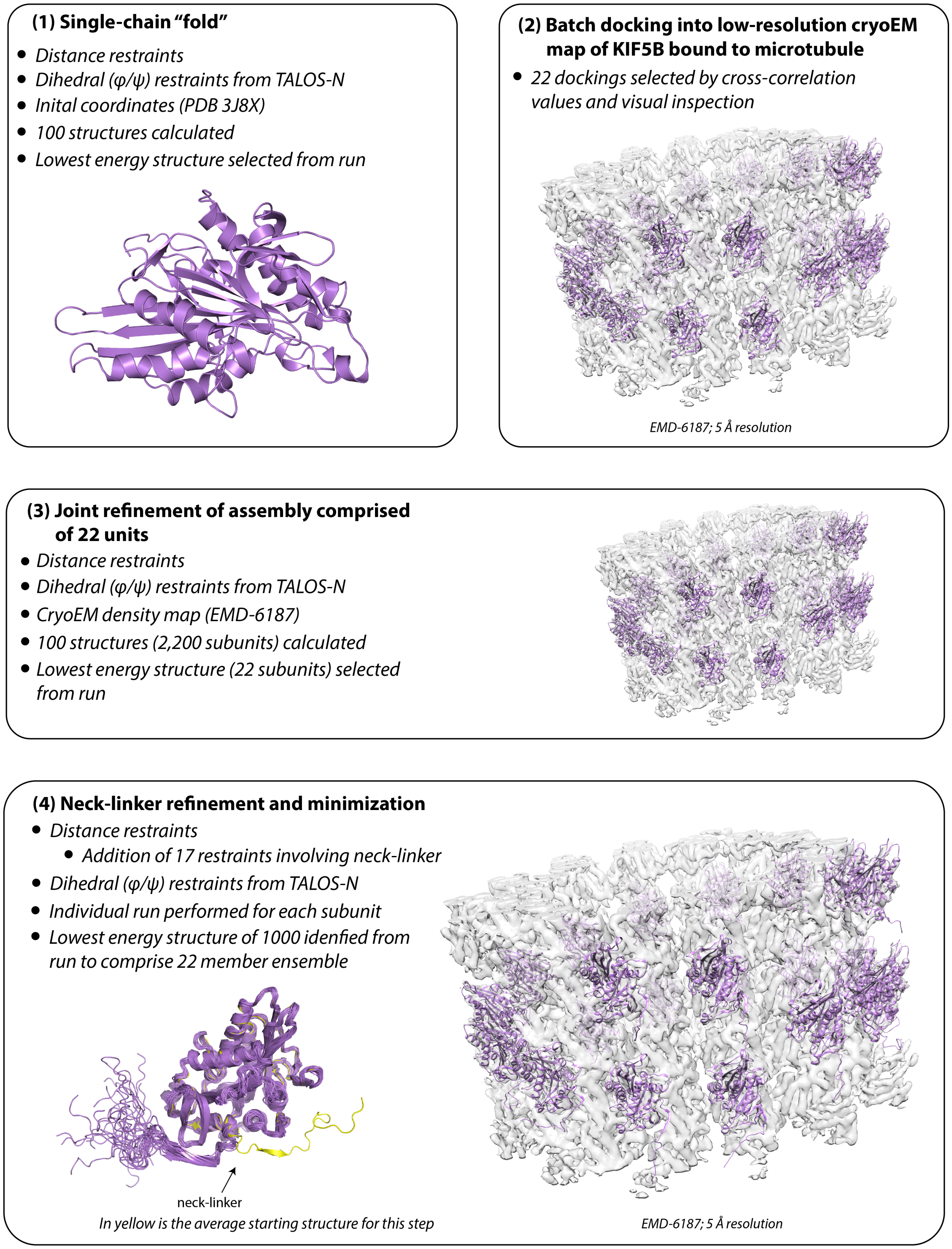

Supplementary Fig. 8. Flowchart of the structure determination protocol for KIF5B bound to MTs.

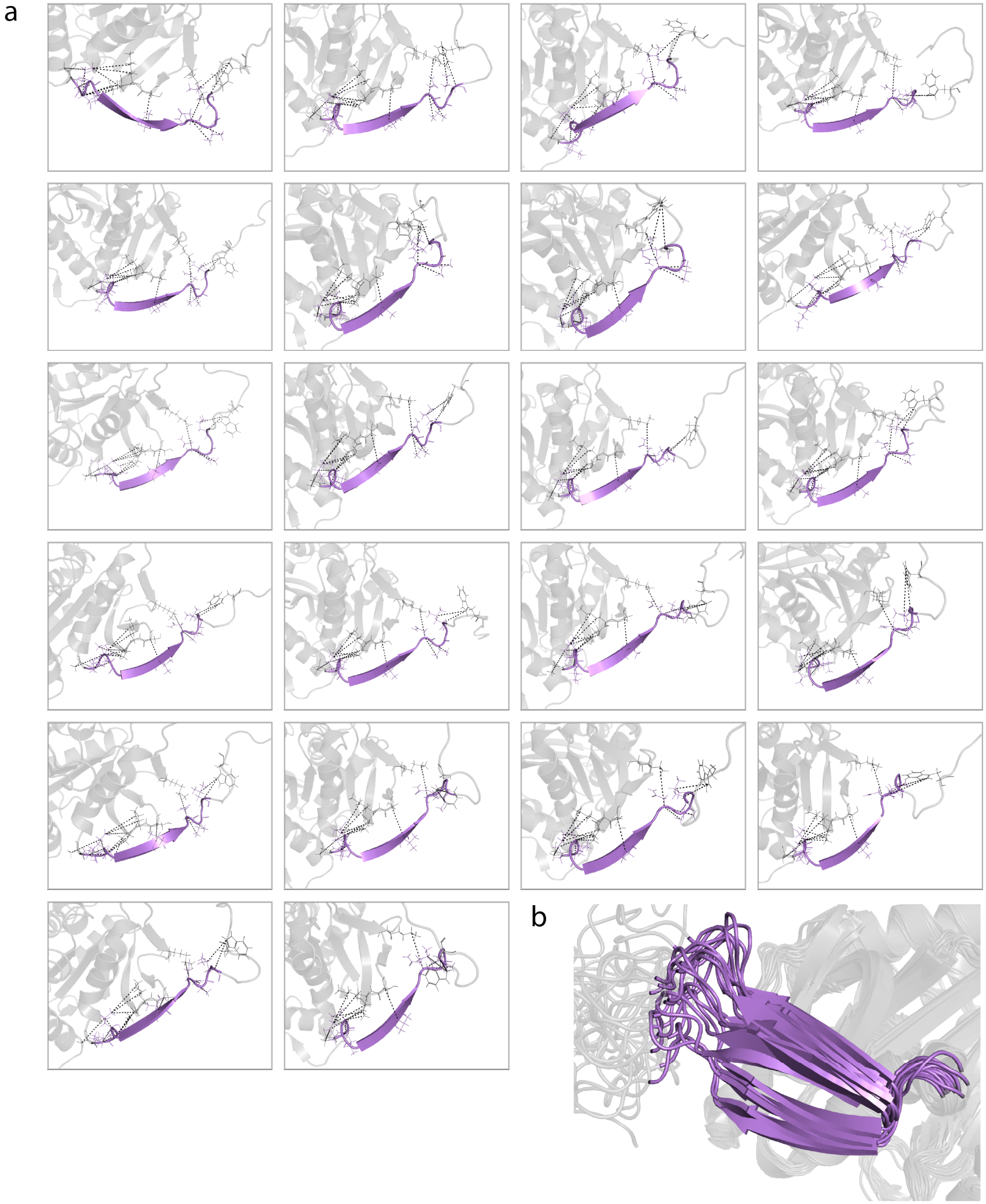

Supplementary Fig. 9. Neck-linker conformations of the 22-member ensemble, shown in **a** individual subunit in the order of lowest to highest energy, and **b** superposition of all structures.

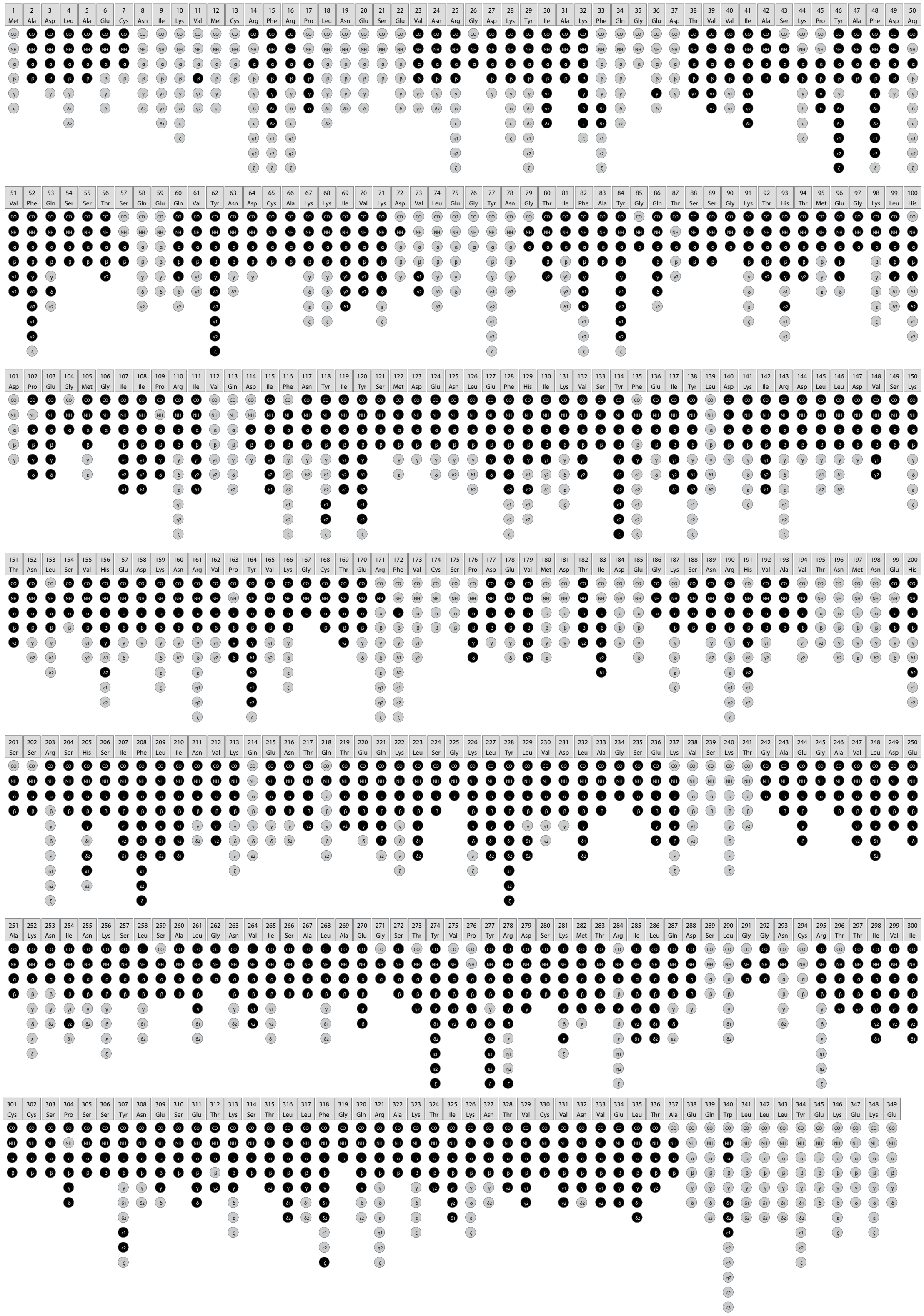

Supplementary Fig. 10. Graphical summary of resonance assignments of KIF5B bound to MTs. Atoms with assigned resonances are shown in black circles and those without assigned resonances are shown in gray circles.

Supplementary Table 1 Acquisition and processing parameters for solid-state NMR experiments

Experiments	Acquisition			Processing		
	ω3	ω2	ω1	ω3	ω2	ω1
14.1 T, 3.2 mm rotor, U-^{13}C,^{15}N-Kif5b/MT ~7.5 mg Kif5b out of ~82.4 mg K/MT Complex						
CORD (τ_{mix} = 50 ms)		2272 complex; SW = 45.5 kHz 36 scans	840 real; (States-TPPI) SW = 28.0 kHz		45/60/90-degree sinebell; Lorentzian-to-Gaussian transformation	45/60/90-degree sinebell; Lorentzian-to-Gaussian transformation
2D NCACX		1818 complex; SW = 45.5 kHz 256 scans	80 real; (States-TPPI) SW = 3.5 kHz		60/90-degree sinebell; Lorentzian-to-Gaussian transformation	Forward linear prediction of 40 points; 60/90-degree sinebell; Lorentzian-to-Gaussian transformation
3D NCACX	1818 complex; SW = 45.5 kHz 48 scans	64 real; (States-TPPI) SW = 6.0 kHz	50 real; (States-TPPI) SW = 2.4 kHz	90-degree sinebell; Lorentzian-to-Gaussian transformation	90-degree sinebell; Lorentzian-to-Gaussian transformation	90-degree sinebell; Lorentzian-to-Gaussian transformation
3D NCOCX	1818 complex; SW = 45.5 kHz 96 scans	36 real; (States-TPPI) SW = 3.5 kHz	50 real; (States-TPPI) SW = 2.4 kHz	90-degree sinebell; Lorentzian-to-Gaussian transformation	90-degree sinebell; Lorentzian-to-Gaussian transformation	90-degree sinebell; Lorentzian-to-Gaussian transformation
20.0 T, 3.2 mm rotor, U-^{13}C,^{15}N-Kif5b/MT ~5.0 mg Kif5b out of ~54.5 mg K/MT Complex						
CORD (τ_{mix} = 50 ms)		2048 complex; SW = 64.1 kHz 192 scans	380 real; (States-TPPI) SW = 42.8 kHz		45/60/90-degree sinebell; Lorentzian-to-Gaussian transformation	Forward linear prediction of 380 points; 45/60/90-degree sinebell; Lorentzian-to-Gaussian transformation
2D NCACX		2048 complex; SW = 64.1 kHz 3072 scans	78 real; (States-TPPI) SW = 4.3 kHz		60/90-degree sinebell; Lorentzian-to-Gaussian transformation	Forward linear prediction of 78 points; 60/90-degree sinebell; Lorentzian-to-Gaussian transformation
3D NUS NCACX	2048 complex; SW =64.1 kHz 1792 scans	10 real; (States) SW = 6.0 kHz	12 real; (States) SW = 3.3 kHz	90-degree sinebell; Lorentzian-to-Gaussian transformation	90-degree sinebell; Lorentzian-to-Gaussian transformation	90-degree sinebell; Lorentzian-to-Gaussian transformation
3D NUS NCOCX	2048 complex; SW = 64.1 kHz 2176 scans	11 real; (States) SW = 7.0 kHz	11 real; (States) SW = 3.3 kHz	90-degree sinebell; Lorentzian-to-Gaussian transformation	90-degree sinebell; Lorentzian-to-Gaussian transformation	90-degree sinebell; Lorentzian-to-Gaussian transformation

Experiments	Acquisition			Processing		
	ω3	ω2	ω1	ω3	ω2	ω1
14.1 T, 3.2 mm rotor, [2-^{13}C],[U-^{15}N]-Kif5b/MT ~4.2 mg Kif5b out of ~55.0 mg K/MT Complex						
CORD (τ_{mix} = 200 ms)		2048 complex; SW = 45.5 kHz 256 scans	512 real; (States-TPPI) SW = 28.0 kHz		45/60/90-degree sinebell; Lorentzian-to-Gaussian transformation	45/60/90-degree sinebell; Lorentzian-to-Gaussian transformation
CORD (τ_{mix} = 500 ms)		2048 complex; SW = 45.5 kHz 256 scans	512 real; (States-TPPI) SW = 28.0 kHz		45/60/90-degree sinebell; Lorentzian-to-Gaussian transformation	45/60/90-degree sinebell; Lorentzian-to-Gaussian transformation
20.0 T, 3.2 mm rotor, [1,6-^{13}C],[U-^{15}N]-Kif5b/MT ~4.5 mg Kif5b out of ~55.0 mg K/MT Complex						
CORD (τ_{mix} = 50 ms)		2048 complex; SW = 64.1 kHz 480 scans	320 real; (States-TPPI) SW = 40.0 kHz		45/60/90-degree sinebell; Lorentzian-to-Gaussian transformation	Forward linear prediction of 320 points; 45/60/90-degree sinebell; Lorentzian-to-Gaussian transformation
CORD (τ_{mix} = 200 ms)		2048 complex; SW = 64.1 kHz 600 scans	320 real; (States-TPPI) SW = 40.0 kHz		45/60/90-degree sinebell; Lorentzian-to-Gaussian transformation	Forward linear prediction of 320 points; 45/60/90-degree sinebell; Lorentzian-to-Gaussian transformation
CORD (τ_{mix} = 500 ms)		2048 complex; SW = 64.1 kHz 640 scans	320 real; (States-TPPI) SW = 40.0 kHz		45/60/90-degree sinebell; Lorentzian-to-Gaussian transformation	Forward linear prediction of 320 points; 45/60/90-degree sinebell; Lorentzian-to-Gaussian transformation

Experiments	Acquisition			Processing		
	ω3	ω2	ω1	ω3	ω2	ω1
20.0 T, 1.9 mm rotor, U-^{13}C,^{15}N-Kif5b/MT ~1.8 mg Kif5b out of ~16.1 mg K/MT Complex						
CORD (τ_{mix} = 50 ms)		2048 complex; SW = 64.1 kHz 512 scans	400 real; (States-TPPI) SW = 40.0 kHz		60/90-degree sinebell; Lorentzian-to-Gaussian transformation	Forward linear prediction of 380 points; 60/90-degree sinebell; Lorentzian-to-Gaussian transformation
20.0 T, 1.9 mm rotor, U-^{13}C,^{15}N-ADP-Kif5b/MT ~1.9 mg Kif5b out of ~16.0 mg K/MT Complex						
CORD (τ_{mix} = 50 ms)		2048 complex; SW = 64.1 kHz 400 scans	400 real; (States-TPPI) SW = 45.0 kHz		60/90-degree sinebell; Lorentzian-to-Gaussian transformation	Forward linear prediction of 380 points; 60/90-degree sinebell; Lorentzian-to-Gaussian transformation
20.0 T, 1.9 mm rotor, U-^{13}C,^{15}N-Kif5b/MT ~1.1 mg Kif5b out of ~10.0 mg K/MT Complex						
NH-HETCOR		1024 complex; SW = 34.5 kHz 128 scans	128 real; (States-TPPI) SW = 45.0 kHz		45/60-degree sinebell; Lorentzian-to-Gaussian transformation	45/60-degree sinebell; Lorentzian-to-Gaussian transformation
20.0 T, 1.3 mm rotor, U-^{13}C,^{15}N-Kif5b/MT ~0.6 mg Kif5b out of ~4.4 mg K/MT Complex						
NH-HETCOR		1024 complex; SW = 34.5 kHz 192 scans	128 real; (States-TPPI) SW = 45.0 kHz		45/60-degree sinebell; Lorentzian-to-Gaussian transformation	45/60-degree sinebell; Lorentzian-to-Gaussian transformation

Supplementary Table 2 Assigned solid-state chemical shifts of Kif5b in complex with microtubules

Residue	N	C’	Cβ	Cα	Cγ	Cδ	Cε	Cζ
A2	130.6	177.2	51.6	18.1				
D3	118.8	175.6	56.2	41.7				
L4	117.5	175.9	52.3	39.8				
A5	123.7	175.3	50.5	21.7				
E6	119.2	177.7	58.4	29.5	34.9	183.2		
C7	118.1	172.2	56.1	31.2				
V11				31.8				
R14		174.3	54.6					
F15	126.8	176.1	61.0	40.2	138.2	132.7		
R16	122.4	176.0	54.3	31.4				
P17			61.3	32.7	28.1	51.2		
V23	120.5	176.8	65.3	31.6				
N24	119.1	177.5	55.5	37.8				
R25	119.0	175.5	57.0	31.1				
G26	111.0		46.7					
D27	119.9	175.7	55.6	39.9				
K28	121.6	174.3	54.5	34.6				
Y29	124.9	174.6	60.2	41.6				
I30	127.1	174.9	60.2	37.8	27.3/18.0	14.0		
A31	123.1	175.5	51.1	20.3				
K32	119.2	174.9	55.1	34.0	24.9	29.0	41.8	
F33						132.9		
E36					36.6			
T38	115.7	174.3	60.0	71.6	20.3			
V39	119.9	174.1	58.9	37.0				
V40	121.8	175.4	60.8	31.8				
I41	126.4	176.5	58.7	40.0	26.8/16.7	11.9		
A42	136.0	176.4	53.2	16.4				
S43	117.4		60.6	62.9				
K44	120.7		52.3	34.1	23.9	27.1		
P45			60.8	31.9	26.3	50.3		
Y46	120.7	172.2	57.0	39.8		133.3	117.7	156.5
A47	126.4	174.9	50.2	20.9				
F48	121.5	175.0	57.7	40.6	140.0	132.4	131.0	

D49	119.4	175.8	56.9	42.0			
R50	116.0	172.7	57.1				
V51	126.9	174.2	60.5	33.3	22.6/20.8		
F52	126.9	175.5	55.3	39.6	138.7	132.8	130.6
Q53	122.2	176.9	55.0	29.0		179.0	
S54	117.1	177.2	61.9	63.6			
S55	112.8	176.5	57.8	62.9			
T56	120.5	174.9	64.5	68.7	22.5		
S57			57.4	64.8			
Q60	118.7	177.6	58.7	29.0	33.7		
V61	118.8	177.0	66.8	31.2	22.1		
Y62	121.2	177.7	60.4	38.9		132.8	
N63	117.1	177.3	56.8	39.8			
D64	118.4	176.5	58.1	41.5			
C65	114.1	175.4	61.1	29.5			
A66	120.6	177.3	53.0	18.9			
K67	117.5	177.4	58.6	31.6			
K68	119.0	178.1	57.4	31.2			
I69	120.4	176.9	65.5	37.8	30.6/17.2	13.7	
V70	118.7	177.0	66.8	31.2	22.1		
K71	119.4	178.4	59.8	32.8	24.7	28.6	
D72	115.7						
V73					23.2		
L74			58.0	42.4	27.1	22.5/25.1	
G79	108.4	172.6	45.2				
T80	115.6	173.5	62.4	71.5	20.3		
I81	128.9	176.1	60.6	39.8			
F82	122.2	172.4	55.3	42.5	138.8	131.7	
A83	130.6	175.2	51.6	18.1			
Y84	121.6	172.2	56.0	40.0	130.6	132.1	117.6
G85	113.2		45.3				
Q86	116.0	175.8	54.4	30.3	32.9	179.7	
T87		177.6	65.2	68.3	22.1		
S88	117.5	174.7	57.2	64.5			
S89	115.4	173.9	59.3	64.9			
G90	109.1	172.8	45.0				
K91	121.3	177.4	60.6	31.9			

T92	122.2	177.5	66.1	67.8	21.4		
H93	121.2	176.6	60.5	31.3	132.7	119.7	
T94	113.3	175.4	66.3	68.2	20.4		
M95	118.4	178.1	57.4				
E96	120.2	177.2	57.2	29.3			
G97	108.5	172.6	45.1				
K98	122.1	176.7	54.3				
L99	125.2	176.8	55.7	41.5	27.4		
H100	117.9		57.4	32.1	131.8	118.1	137.9
P102		177.3	64.9	31.4	26.9	49.8	
E103	118.6	178.6	57.7	30.4	36.1	180.6	
G104	106.4		44.5				
M105	119.3	174.2	54.3	34.4			
G106	106.4	174.2	44.1				
I107	117.9	176.9	66.4	38.8	29.8/17.1	12.7	
I108	117.1	175.1	68.2	34.5	30.1/16.1	12.7	
P109		178.4	65.2	30.0	28.5	48.8	
R110	119.0	177.7	58.6	29.0			
I111	120.7	176.7	65.9	37.7	29.0/15.4	13.4	
V112			66.5	31.4	24.3/20.8		
I115	123.2	177.7	64.6	37.1	29.3/16.4	12.7	
F116	116.7		61.2	39.4			
N117	115.8	176.5	53.5	38.8			
Y118	120.4	178.1	58.7	40.3			117.7
I119	119.8	176.9	64.6	38.3	29.7/17.1	12.6	
Y120	114.9	178.3	59.9	38.3	129.2	133.4	116.7
S121	114.9	174.2	57.9	62.6			
M122	120.9	177.8	55.0	33.7			
D123	122.3	176.1	53.7	42.6			
E124	120.3	176.9	57.8	29.5			
N125	119.9	173.9	55.5	37.8			
L126	123.7	175.0	54.8	41.6	26.4	22.0	
E127	122.0	176.0	54.5	31.7	35.9	183.7	
F128	125.1	174.3	57.5	39.8	137.4	132.1	
H129	123.2	173.8	53.7	32.4	131.7	118.6	
I130	127.6	174.1	60.7	36.9	27.7/18.4	14.8	
K131	126.8	174.9	54.6	36.1			

V132	122.5	174.9	57.7	34.7	22.5			
S133	119.5	172.3	56.8	65.8				
Y134	122.3	173.6	57.2	40.5	130.5	134.3	118.5	158.5
F135	123.9		57.3		141.6			
E136	123.6	174.6	54.3	33.0				
I137	123.7	174.4	59.4	40.9	28.5/16.2	14.6		
Y138	125.6	174.4	57.2	40.6	129.6	131.7		
D140	114.2	177.2	55.7	40.8				
K141	117.6	177.3	58.6	31.6				
I142	121.6	174.9	61.3	39.4	28.1/19.0	13.9		
R143	126.6	175.2	54.7	31.3				
D144	122.1	177.2	54.4	41.2				
L145	125.0	178.3	57.3	39.8				
L146	119.0	179.1	56.6	42.0				
D147	120.2	175.8	53.3	40.2				
V148	117.6	176.4	62.7	30.7	22.4/20.5			
S149	115.8	172.3	56.9	65.7				
K150	121.8	176.5	55.0	34.3	24.0	28.7	42.5	
T151	116.3	176.2	61.4	71.8	21.6			
N152	117.7	173.9	52.5	40.0				
L153	122.9		55.0	42.5				
S154	117.1	172.5	56.6	63.8				
V155	126.2	176.2	61.2	31.7				
H156	123.5	174.0	54.3	33.4	132.2	120.4		
E157	124.3	178.5	59.3	29.5				
D158	123.3	176.6	55.1	39.6				
K159	120.2	178.5	59.8	32.7				
N160	115.8	176.8	53.3	38.3				
R161	117.3	175.1	57.5	27.6				
V162	119.7	174.4	61.8	33.8				
P163		176.4	62.7	32.9	27.9	51.2		
Y164	119.1	172.6	56.6	42.0	130.1	131.9	118.0	
V165	121.7	173.1	60.6	31.9				
K166	128.6	176.3	57.9	32.6				
G167	115.0	174.0	45.3					
C168	116.7	174.2	57.5	27.5				
T169	118.7	175.4	63.7	68.5	21.5			

E170	120.7	174.4	57.0	29.2				
R171	123.8							
F172			58.9					
P176			65.1	31.9	27.1	50.6		
D177	120.4	177.4	57.0	40.5				
E178	119.0	177.6	58.7	31.5				
V179	118.9	177.0	66.2	31.5	22.7/21.4			
T182	113.4	175.4	66.3	68.1	20.4			
I183			60.1	39.8	27.2/16.8	13.2		
G186	107.0	174.7	47.1					
K187	119.8		58.6	33.7				
S188	114.1	175.5	61.1	63.0				
N189	119.6	175.3	56.0	37.8				
R190	118.4	177.2	56.4	31.2				
H191	121.4		55.6	27.2	132.2	120.2		
V192	121.1	176.3	60.9	31.8				
A193	135.4	176.4	53.1	16.5				
V194	117.4		59.3	31.4	22.2			
E199		176.5	59.0	30.3				
H200	121.2	176.6	60.7	31.9	132.0	120.2		
S201	113.2	172.3	60.0	62.1				
S202	116.5	175.4	59.9	63.5				
R203	118.9	175.6	55.5					
S204	111.3	173.0	57.0	66.6				
H205	123.8	174.0	54.2	30.0	131.9	118.3	138.4	
S206	122.2	172.6	56.6	63.8				
I207	123.5	175.7	60.6	39.8	27.5/16.9	14.0		
F208	121.9	173.1	56.8	40.9	139.6	132.4	131.2	128.6
L209	124.5	174.3	52.7	42.7	26.8	25.0		
I210	123.7	174.2	59.6	36.8	28.7/18.3	14.8		
N211	124.6	174.3	52.6	40.8				
V212	125.5	174.3	61.6	32.0	20.5			
K213	130.6	174.3	54.8	34.4				
E215	118.8	175.5	56.2	29.7				
N216	125.2	174.7	52.2	39.7				
T217	121.5	175.8	63.9	68.5	21.6			
Q218	120.6							

T219	110.4	174.2	62.6	69.1	22.4			
E220	116.7	174.7	57.5	30.0	36.6			
Q221	122.5	175.4	55.1	29.1	31.9	179.8		
K222	120.7	174.7	55.2	36.3				
L223	122.4	176.1	53.6	46.1	26.6	24.9/23.6		
S224	117.5	172.5	56.6	63.8				
G225	112.7	172.8	45.0					
K226	121.7	173.9	54.0	34.4	25.0	28.6		
L227	127.5	174.6	53.6	42.1	27.3	23.7		
Y228	124.8	174.0	55.8	41.5	129.5	132.7	118.3	158.0
L229	124.8	174.3	54.0	43.4		24.9		
V230	124.3	174.1	60.9	32.5				
D231	126.4	174.8	53.2	41.9				
L232	122.9	177.1	54.9	42.5	26.8	25.0		
A233	123.0	175.6	51.1	20.3				
G234	103.9	173.7	44.2					
S235	117.2	173.6	58.0	64.4				
E236	118.2	176.7	57.0	32.1	35.9			
K237	119.2	175.1	56.7	31.2	25.0	28.3		
V238	123.2	177.6	64.6	31.1	22.2/19.8			
S239	115.8	177.0	61.3	63.1				
G242	108.9	175.3	46.6					
A243	121.0	176.8	53.4	18.9				
E244	118.5	178.5	55.7	29.6	36.3	183.4		
G245	106.2	176.3	47.1					
A246	121.6	177.4	55.3	17.8				
V247	119.0	175.6	67.3	31.2	22.3			
L248	123.0	175.4	55.1	39.6	26.9	24.0/21.6		
D249	122.0	176.3	54.3	41.7	178.8			
E250	121.4	174.0	60.3	31.8	35.8	183.3		
A251	124.6	176.5	54.7	18.0				
K252	115.9	175.1	55.1	32.9	26.0	28.2		
N253	119.5	176.1	55.4					
I254	119.8	176.9	65.1	37.8	29.6/17.2	14.6		
N255	119.6	178.2	55.4	37.8				
K256	121.3	177.1	60.9	31.9				
S257	114.0	175.3	61.1	63.0				

L258	121.0	175.0	57.0	39.9				
S259	116.3	176.9	61.8	63.1				
A260	124.1	178.9	54.7	17.9				
L261	120.4	178.3	57.7	40.6	25.5			
G262	107.1	174.6	46.6					
N263	120.2	177.4	57.4	38.9				
V264	119.4	177.0	67.1	30.9	23.2/21.4			
I265	117.2	178.6	64.7	37.2	29.5/16.3	12.7		
S266	117.3	176.4	62.7	63.7				
A267	125.4	180.5	54.8	18.0				
L268	120.4	179.7	57.6	40.5				
A269	121.3	179.1	55.1	18.1				
E270	115.5	176.7	58.8	30.3	36.4	183.2		
G271	106.4	174.8	45.6					
S272	116.7	175.1	57.3	63.4				
T273	119.6		65.2	68.3	22.1			
Y274	118.9	174.0	55.5	41.6	129.3	132.7	117.9	156.8
V275	128.7		57.8	34.9	22.6			
P276			62.3	31.4	27.1	50.2		
Y277	118.5	177.5	58.5	41.4	129.6	131.1	119.2	158.4
R278	117.1	176.5	57.9	29.9	27.9	43.4		159.0
D279	121.2	175.8	55.5	37.1	178.7			
S280	111.6	172.3	56.8	66.4				
K281	122.0	176.5	54.5	31.7	24.9		41.2	
M282	116.2	176.9	59.8	34.2	30.4		17.9	
T283	104.7	174.6	64.7	67.7	22.5			
R284	119.5		58.6	29.7	28.0			
I285	120.6	176.6	66.0	38.4	29.0/18.5	12.6		
L286	116.7	175.8	53.3	40.3	26.7	24.5/21.5		
Q287	121.6	176.7	59.8	29.4	32.0	179.6		
D288	123.4	176.7	54.9	39.6				
G291	104.8		46.5					
G292	113.5	174.7	45.6					
N293	121.1							
R295	125.5	174.7	55.1	29.2				
T296	122.5	172.7	60.9	72.7	22.1			
T297	124.2	173.1	60.4	71.7	24.2			

I298	127.3	174.6	60.1	39.8	27.2/16.8	14.0		
V299	127.4	174.3	60.7	33.4	20.9/20.0			
I300	123.9	174.2	60.6	39.2	28.5/16.9	14.0		
C301	123.4	174.0	58.0	29.6				
C302	118.9	173.7	57.3	31.0				
S303	118.5	173.5	55.6	63.8				
P304		176.6	62.6	30.9	26.4	50.0		
S305	115.7	176.6	56.9	65.5				
S306	115.2	175.4	61.1	63.1				
Y307	121.1	176.8	60.7	38.9	129.3	133.5	119.2	
N308	112.8	174.3	52.4	38.7				
E309	121.2	177.0	60.8	29.4	35.5			
S310	114.0	175.3	61.1	63.0				
E311	120.6	177.6	58.4	29.6	36.4	183.2		
T312	117.5	177.0	66.8	68.1	22.3			
K313	118.5	178.2	59.6	31.5	24.5	28.8		
S314	115.8	177.0	61.3	63.1				
T315	117.5	177.0	66.8	68.1	22.3			
L316	118.5	177.9	58.7	40.1	27.0	24.6/21.4		
L317	119.3	177.8	58.6	41.2	28.0			
F318	120.4	174.5	61.8	38.3	139.1	131.2		129.6
G319	105.1	176.6	47.3					
Q320	119.5	178.4	59.6	29.5	32.9			
R321	117.2	177.5	58.6	31.6				
A322	122.3	177.1	55.3	19.4				
K323	117.1	177.5	57.2	33.9				
T324	108.4	175.5		69.9	21.8			
I325	120.9	175.8	61.9	38.6	25.0/18.5	14.1		
K326	121.3	174.7	55.1	37.1				
N327	122.4	174.0	55.4	39.8				
T328	118.5	175.4	61.0	69.9	21.2			
V329	120.4	176.5	59.0	35.5	22.4/19.3			
C330	117.1	174.2	57.9	30.0				
V331	120.6	177.0	61.9	32.2	21.3/20.5			
N332	124.7	173.9	52.6	38.4	176.1			
V333	120.8	176.0	65.4	31.6	20.9			
E334	118.3	175.1	56.5	31.2	36.3	182.4		

L335	122.9	175.6	55.5	42.4	26.5	24.9
T336	113.2	174.2	60.8	71.8	20.5	
A337	124.0		54.8	18.2		
W340	120.5		62.1			126.5
