# Supplementary material for: Magic-angle-spinning NMR structure of the kinesin-1 motor domain assembled with microtubules reveals the elusive neck linker orientation
Source: Nat Commun. 2022 Nov 10;13:6795. doi: 10.1038/s41467-022-34026-w (PMC9649657; doi:10.1038/s41467-022-34026-w)
Supplement: Supplementary file 2 — Reporting Summary [file 41467_2022_34026_MOESM2_ESM.pdf]

nature portfolio

Corresponding author(s): Tatyana Polenova, Angela M. Gronenborn, John C. Williams

Last updated by author(s): 10/03//22

# Reporting Summary

Nature Portfolio wishes to improve the reproducibility of the work that we publish. This form provides structure for consistency and transparency in reporting. For further information on Nature Portfolio policies, see our Editorial Policies and the Editorial Policy Checklist.

## Statistics

For all statistical analyses, confirm that the following items are present in the figure legend, table legend, main text, or Methods section.

| n/a | Confirmed | |
|---|---|---|
| ☐ | ☒ | The exact sample size (*n*) for each experimental group/condition, given as a discrete number and unit of measurement |
| ☒ | ☐ | A statement on whether measurements were taken from distinct samples or whether the same sample was measured repeatedly |
| ☒ | ☐ | The statistical test(s) used AND whether they are one- or two-sided<br>*Only common tests should be described solely by name; describe more complex techniques in the Methods section.* |
| ☒ | ☐ | A description of all covariates tested |
| ☒ | ☐ | A description of any assumptions or corrections, such as tests of normality and adjustment for multiple comparisons |
| ☒ | ☐ | A full description of the statistical parameters including central tendency (e.g. means) or other basic estimates (e.g. regression coefficient) AND variation (e.g. standard deviation) or associated estimates of uncertainty (e.g. confidence intervals) |
| ☒ | ☐ | For null hypothesis testing, the test statistic (e.g. *F*, *t*, *r*) with confidence intervals, effect sizes, degrees of freedom and *P* value noted<br>*Give P values as exact values whenever suitable.* |
| ☒ | ☐ | For Bayesian analysis, information on the choice of priors and Markov chain Monte Carlo settings |
| ☒ | ☐ | For hierarchical and complex designs, identification of the appropriate level for tests and full reporting of outcomes |
| ☒ | ☐ | Estimates of effect sizes (e.g. Cohen's *d*, Pearson's *r*), indicating how they were calculated |

*Our web collection on statistics for biologists contains articles on many of the points above.*

## Software and code

Policy information about availability of computer code

| | |
|---|---|
| Data collection | NMR data were collected using Bruker TopSpin version 3.5 and 4.0, a standard commercial program. |
| Data analysis | MAS NMR data were processed with Bruker TopSpin version 3.2 and NMRPipe (version mac). NMR spectra were visualized and analyzed using NMRFAM-Sparky version 1.470-3.190 and CcpNmr Analysis version 2.4.2. Backbone dihedral angel restraints were generated from experimental NMR data using TALOS-N version 4.12. MAS NMR structures of the single KIF5B, seeded from the coordinates of the cryo-EM structure (PDB 3J8X), were calculated and refined in the Xplor-NIH 2.53.<br><br>Docking calculations and coordinate transformation to generate the structure of the KIF5B/MT assemblies were accomplished with in-house UCSF Chimera python scripts. (UCSF Chimera version 1.13)<br><br>Restraint tallying and format conversions were carried out with in-house Python 2.7 scripts. Structure ensembles were rendered for visualization in PyMOL 1.8.6.2 using in-house shell/bash scripts for batch rendering. Renders of structures docked into the cryo-EM density were performed with UCSF Chimera 1.13 with in-house UCSF Chimera python scripts. Secondary structure elements were classified according to TALOS-N and manual inspection. |

For manuscripts utilizing custom algorithms or software that are central to the research but not yet described in published literature, software must be made available to editors and reviewers. We strongly encourage code deposition in a community repository (e.g. GitHub). See the Nature Portfolio guidelines for submitting code & software for further information.

## Data

Policy information about availability of data

All manuscripts must include a data availability statement. This statement should provide the following information, where applicable:

- Accession codes, unique identifiers, or web links for publicly available datasets
- A description of any restrictions on data availability
- For clinical datasets or third party data, please ensure that the statement adheres to our policy

The coordinates for the all atom NMR structures have been deposited in the Protein Data Bank under accession codes PDB 7RIK [http://doi.org/10.2210/pdb7RIK/pdb]. The MAS NMR chemical shifts have been deposited in the Biological Magnetic Resonance Data Bank under accession code 30936 [https://dx.doi.org/10.13018/BMR30936]. The Cryo-EM density used in this study are available in the EM data bank and Protein Data Bank under accession codes EMD-6187 [http:// www.ebi.ac.uk/pdbe/entry/emdb/EMD-6187] and PDB 3J8X [http://doi.org/10.2210/pdb3J8X/pdb]. Source Data are provided with this paper.

# Field-specific reporting

Please select the one below that is the best fit for your research. If you are not sure, read the appropriate sections before making your selection.

☒ Life sciences ☐ Behavioural & social sciences ☐ Ecological, evolutionary & environmental sciences

For a reference copy of the document with all sections, see nature.com/documents/nr-reporting-summary-flat.pdf

# Life sciences study design

All studies must disclose on these points even when the disclosure is negative.

| | |
|---|---|
| Sample size | The sample size for MAS NMR experiments were chosen according to the size and capacity of magic-angle spinning rotors. Multiple samples of isotopically labeled recombinant protein were prepared and packed into rotors of different sizes. |
| Data exclusions | No data were excluded from the analyses. |
| Replication | The measurements were replicated at least three times with similar results. |
| Randomization | This study does not include any clinical trial or research. |
| Blinding | This study does not include any clinical trial or research. |

# Reporting for specific materials, systems and methods

We require information from authors about some types of materials, experimental systems and methods used in many studies. Here, indicate whether each material, system or method listed is relevant to your study. If you are not sure if a list item applies to your research, read the appropriate section before selecting a response.

## Materials & experimental systems

| n/a | Involved in the study | |
|---|---|---|
| ☒ | ☐ | Antibodies |
| ☒ | ☐ | Eukaryotic cell lines |
| ☒ | ☐ | Palaeontology and archaeology |
| ☒ | ☐ | Animals and other organisms |
| ☒ | ☐ | Human research participants |
| ☒ | ☐ | Clinical data |
| ☒ | ☐ | Dual use research of concern |

## Methods

| n/a | Involved in the study | |
|---|---|---|
| ☒ | ☐ | ChIP-seq |
| ☒ | ☐ | Flow cytometry |
| ☒ | ☐ | MRI-based neuroimaging |
